# Supplementary material for: Retrospective survey of youth sports participation: Development and assessment of reliability using school records
Source: PLoS One. 2021 Sep 17;16(9):e0257487. doi: 10.1371/journal.pone.0257487 (PMC8448309; doi:10.1371/journal.pone.0257487)
Supplement: S1 Survey — (PDF) [file pone.0257487.s001.pdf]

Introduction

Thank you for taking the time to complete this survey.

Please enter your name and e-mail address below.

First Name

Last Name

Maiden Name

E-mail Address

Please select your current age.

▼

Please select your gender.

- Male
- Female

Please select the primary race you identify with.

- American Indian or Alaska Native
- Asian
- Black or African American
- Native Hawaiian or Other Pacific Islander
- White
- Prefer not to answer

Please select the primary ethnicity you identify with.

- Hispanic or Latino
- Not Hispanic or Latino
- Prefer not to answer

Please enter the name of the High School you attended.

If you attended more than one High School, please enter the High School at which you spent more time.

In what year did you graduate High School?

Now we're going to ask you questions related to sports participation.

Grades K-6

Please select all sports that you participated in on an organized team at any time from Kindergarten through 6th Grade.

If a sport you participated in is not listed, you may select "Other Sport" and type in up to three other sports.

If you did not participate in any sports, please select "None."

- ☐ None
- ☐ American Flag Football
- ☐ American Tackle Football
- ☐ Baseball
- ☐ Basketball

- ☐ Cheerleading
- ☐ Cross Country
- ☐ Field Hockey
- ☐ Golf
- ☐ Gymnastics
- ☐ Ice Hockey
- ☐ Lacrosse
- ☐ Rugby
- ☐ Soccer
- ☐ Softball
- ☐ Swimming
- ☐ Tennis
- ☐ Track & Field
- ☐ Volleyball
- ☐ Water Polo
- ☐ Wrestling
- ☐ ______ Other Sport 1
- ☐ ______ Other Sport 2
- ☐ ______ Other Sport 3

Did you have any physical limitations that prevented you from playing on an organized sports team from Kindergarten through Grade 6?

- ○ Yes
- ○ No
- ○ I prefer not to answer

Note. The following questions are in regards to your participation in sports from Kindergarten through Grade 6.

In which grades did you play baseball? You may provide your best estimate if you are not exactly sure.

- ☐ Kindergarten
- ☐ Grade 1
- ☐ Grade 2
- ☐ Grade 3
- ☐ Grade 4
- ☐ Grade 5
- ☐ Grade 6

Please select all that apply in regards to your participation in baseball. You may provide your best estimate if you are not exactly sure.

- ☐ My team traveled for games or tournaments.
- ☐ My team participated in playoffs.
- ☐ My team won a championship.
- ☐ None of the above.

On average, how many hours per week did you spend practicing baseball with the team? You may provide your best estimate if you are not exactly sure.

- ◯ 1-4 hours

- 5-9 hours
- 10-14 hours
- 15-19 hours
- 20+ hours

On average, how many hours per week did you spend playing in baseball games? You may provide your best estimate if you are not exactly sure.

- 1-4 hours
- 5-9 hours
- 10-14 hours
- 15-19 hours
- 20+ hours

Please select all positions you played in. You may provide your best estimate if you are not exactly sure.

- Pitcher
- Catcher
- Baseman/Shortstop
- Outfielder
- Other
- None of the above

Please select all that apply in regards to the types of leagues you played baseball in. You may provide your best estimate if you are not exactly sure.

- ☐ I participated in a competitive, school based league
- ☐ I participated in a competitive, non-school based league
- ☐ I participated in a club, recreational, or intramural league at school
- ☐ I participated in a club, recreational, or intramural league outside of school
- ☐ ________ Other
- ☐ None of the above

In which grades did you play basketball? Please select all that apply. You may provide your best estimate if you are not exactly sure.

- ☐ Kindergarten
- ☐ Grade 1
- ☐ Grade 2
- ☐ Grade 3
- ☐ Grade 4
- ☐ Grade 5
- ☐ Grade 6

Please select all that apply in regards to your participation in basketball. You may provide your best estimate if you are not exactly sure.

- ☐ My team traveled for games or tournaments.
- ☐ My team participated in playoffs.
- ☐ My team won a championship.
- ☐ None of the above.

On average, how many hours per week did you spend practicing basketball with the team? You may provide your best estimate if you are not exactly sure.

- ○ 1-4 hours
- ○ 5-9 hours
- ○ 10-14 hours
- ○ 15-19 hours
- ○ 20+ hours

On average, how many hours per week did you spend playing in basketball games? You may provide your best estimate if you are not exactly sure.

- ○ 1-4 hours
- ○ 5-9 hours
- ○ 10-14 hours
- ○ 15-19 hours
- ○ 20+ hours

Please select all positions you played in. You may provide your best estimate if you are not exactly sure.

- ☐ Guard
- ☐ Forward
- ☐ Center
- ☐ [ ] Other
- ☐ None of the above

Please select all that apply in regards to the types of leagues you played basketball in. You may provide your best estimate if you are not exactly sure.

- ☐ I participated in a competitive, school based league
- ☐ I participated in a competitive, non-school based league
- ☐ I participated in a club, recreational, or intramural league at school
- ☐ I participated in a club, recreational, or intramural league outside of school
- ☐ __________ Other
- ☐ None of the above

In which grades did you participate in cheerleading? Please select all that apply. You may provide your best estimate if you are not exactly sure.

- ☐ Kindergarten
- ☐ Grade 1
- ☐ Grade 2
- ☐ Grade 3
- ☐ Grade 4
- ☐ Grade 5
- ☐ Grade 6

Please select all that apply in regards to your participation in cheerleading. You may provide your best estimate if you are not exactly sure.

- ☐ I traveled for cheerleading tournaments.
- ☐ My team won a cheerleading tournament.

☐ None of the above

On average, how many hours per week did you spend practicing cheerleading? You may provide your best estimate if you are not exactly sure.

○ 1-4 hours
○ 5-9 hours
○ 10-14 hours
○ 15-19 hours
○ 20+ hours

Please select all that apply in regards to the types of leagues you participated in cheerleading. You may provide your best estimate if you are not exactly sure.

☐ I participated in a competitive, school based league
☐ I participated in a competitive, non-school based league
☐ I participated in a club, recreational, or intramural league at school
☐ I participated in a club, recreational, or intramural league outside of school
☐ [ ] Other
☐ None of the above

Please select all that apply in regards to your participation in cross country. You may provide your best estimate if you are not exactly sure.

☐ I traveled for meets or tournaments.

- ☐ I or my team won a tournament.
- ☐ None of the above

In which grades did you participate in cross country? Please select all that apply. You may provide your best estimate if you are not exactly sure.

- ☐ Kindergarten
- ☐ Grade 1
- ☐ Grade 2
- ☐ Grade 3
- ☐ Grade 4
- ☐ Grade 5
- ☐ Grade 6

On average, how many hours per week did you spend practicing cross country with the team? You may provide your best estimate if you are not exactly sure.

- ○ 1-4 hours
- ○ 5-9 hours
- ○ 10-14 hours
- ○ 15-19 hours
- ○ 20+ hours

On average, how many hours per week did you spend participating in cross country meets? You may provide your best estimate if you are not exactly sure.

- 1-4 hours
- 5-9 hours
- 10-14 hours
- 15-19 hours
- 20+ hours

Please select all that apply in regards to the types of leagues you participated in cross country. You may provide your best estimate if you are not exactly sure.

- I participated in a competitive, school based league
- I participated in a competitive, non-school based league
- I participated in a club, recreational, or intramural league at school
- I participated in a club, recreational, or intramural league outside of school
- Other
- None of the above

In which grades did you participate in field hockey? Please select all that apply. You may provide your best estimate if you are not exactly sure.

- Kindergarten
- Grade 1
- Grade 2
- Grade 3
- Grade 4
- Grade 5
- Grade 6

Please select all that apply in regards to your participation in field hockey. You may provide your best estimate if you are not exactly sure.

- ☐ My team traveled for games or tournaments.
- ☐ My team participated in playoffs.
- ☐ My team won a championship.
- ☐ None of the above

On average, how many hours per week did you spend practicing field hockey with the team? You may provide your best estimate if you are not exactly sure.

- ◯ 1-4 hours
- ◯ 5-9 hours
- ◯ 10-14 hours
- ◯ 15-19 hours
- ◯ 20+ hours

On average, how many hours per week did you spend playing in field hockey games? You may provide your best estimate if you are not exactly sure.

- ◯ 1-4 hours
- ◯ 5-9 hours
- ◯ 10-14 hours
- ◯ 15-19 hours
- ◯ 20+ hours

Please select all positions you played in. You may provide your best estimate if you are not exactly sure.

- ☐ Left/right Defense
- ☐ Left/right Wing
- ☐ Center
- ☐ Goalie
- ☐ __________ Other
- ☐ None of the above

Please select all that apply in regards to the types of leagues you played field hockey in. You may provide your best estimate if you are not exactly sure.

- ☐ I participated in a competitive, school based league
- ☐ I participated in a competitive, non-school based league
- ☐ I participated in a club, recreational, or intramural league at school
- ☐ I participated in a club, recreational, or intramural league outside of school
- ☐ __________ Other
- ☐ None of the above

In which grades did you participate in American tackle football? Please select all that apply. You may provide your best estimate if you are not exactly sure.

- ☐ Kindergarten
- ☐ Grade 1

- ☐ Grade 2
- ☐ Grade 3
- ☐ Grade 4
- ☐ Grade 5
- ☐ Grade 6

Please select all that apply in regards to your participation in American tackle football. You may provide your best estimate if you are not exactly sure.

- ☐ My team traveled for games or tournaments.
- ☐ My team participated in playoffs.
- ☐ My team won a championship.
- ☐ None of the above

On average, how many hours per week did you spend practicing American tackle football with the team? You may provide your best estimate if you are not exactly sure.

- ◯ 1-4 hours
- ◯ 5-9 hours
- ◯ 10-14 hours
- ◯ 15-19 hours
- ◯ 20+ hours

On average, how many hours per week did you spend playing in American tackle football games? You may provide your best estimate if you are not exactly sure.

- 1-4 hours
- 5-9 hours
- 10-14 hours
- 15-19 hours
- 20+ hours

Please select all positions you played in. You may provide your best estimate if you are not exactly sure.

- Quarterback
- Running Back
- Fullback
- Offensive Line
- Wide Receiver
- Tight End
- Defensive Line
- Linebacker
- Cornerback
- Safety
- Kicker/Punter
- Participation in kick off return
- Long Snapper
- Other
- None of the above

Please select all that apply in regards to the types of leagues you played American tackle football in. You may provide your best estimate if you are not exactly sure.

- ☐ I participated in a competitive, school based league
- ☐ I participated in a competitive, non-school based league
- ☐ I participated in a club, recreational, or intramural league at school
- ☐ I participated in a club, recreational, or intramural league outside of school
- ☐ [ ] Other
- ☐ None of the above

In which grades did you participate in American flag football? Please select all that apply. You may provide your best estimate if you are not exactly sure.

- ☐ Kindergarten
- ☐ Grade 1
- ☐ Grade 2
- ☐ Grade 3
- ☐ Grade 4
- ☐ Grade 5
- ☐ Grade 6

Please select all that apply in regards to your participation in American flag football. You may provide your best estimate if you are not exactly sure.

- ☐ My team traveled for games or tournaments.
- ☐ My team participated in playoffs.
- ☐ My team won a championship.
- ☐ None of the above

On average, how many hours per week did you spend practicing American flag football with the team? You may provide your best estimate if you are not exactly sure.

- 1-4 hours
- 5-9 hours
- 10-14 hours
- 15-19 hours
- 20+ hours

On average, how many hours per week did you spend playing in American flag football games? You may provide your best estimate if you are not exactly sure.

- 1-4 hours
- 5-9 hours
- 10-14 hours
- 15-19 hours
- 20+ hours

Please select all positions you played in. You may provide your best estimate if you are not exactly sure.

- Quarterback
- Running Back
- Fullback
- Offensive Line
- Wide Receiver
- Tight End

- ☐ Defensive Line
- ☐ Linebacker
- ☐ Cornerback
- ☐ Safety
- ☐ Kicker/Punter
- ☐ Participation in kick off return
- ☐ Long Snapper
- ☐ [ ] Other
- ☐ None of the above

Please select all that apply in regards to the types of leagues you played American flag football in. You may provide your best estimate if you are not exactly sure.

- ☐ I participated in a competitive, school based league
- ☐ I participated in a competitive, non-school based league
- ☐ I participated in a club, recreational, or intramural league at school
- ☐ I participated in a club, recreational, or intramural league outside of school
- ☐ [ ] Other
- ☐ None of the above

In which grades did you participate in golf? Please select all that apply. You may provide your best estimate if you are not exactly sure.

- ☐ Kindergarten
- ☐ Grade 1
- ☐ Grade 2
- ☐ Grade 3

- [ ] Grade 4
- [ ] Grade 5
- [ ] Grade 6

Please select all that apply in regards to your participation in golf. You may provide your best estimate if you are not exactly sure.

- [ ] My team traveled for matches or tournaments.
- [ ] I or my team won a tournament.
- [ ] None of the above

On average, how many hours per week did you spend practicing golf with the team? You may provide your best estimate if you are not exactly sure.

- ○ 1-4 hours
- ○ 5-9 hours
- ○ 10-14 hours
- ○ 15-19 hours
- ○ 20+ hours

On average, how many hours per week did you spend participating in golf matches? You may provide your best estimate if you are not exactly sure.

- ○ 1-4 hours
- ○ 5-9 hours
- ○ 10-14 hours
- ○ 15-19 hours
- ○ 20+ hours

Please select all that apply in regards to the types of leagues you played golf in. You may provide your best estimate if you are not exactly sure.

- ☐ I participated in a competitive, school based league
- ☐ I participated in a competitive, non-school based league
- ☐ I participated in a club, recreational, or intramural league at school
- ☐ I participated in a club, recreational, or intramural league outside of school
- ☐ [ ] Other
- ☐ None of the above

In which grades did you participate in gymnastics? Please select all that apply. You may provide your best estimate if you are not exactly sure.

- ☐ Kindergarten
- ☐ Grade 1
- ☐ Grade 2
- ☐ Grade 3
- ☐ Grade 4
- ☐ Grade 5
- ☐ Grade 6

Please select all that apply in regards to your participation in gymnastics. You may provide your best estimate if you are not exactly sure.

- ☐ My team traveled for meets or tournaments.
- ☐ I or my team won a tournament.
- ☐ None of the above

On average, how many hours per week did you spend practicing gymnastics with the team? You may provide your best estimate if you are not exactly sure.

- ○ 1-4 hours
- ○ 5-9 hours
- ○ 10-14 hours
- ○ 15-19 hours
- ○ 20+ hours

On average, how many hours per week did you spend participating in gymnastics meets?

- ○ 1-4 hours
- ○ 5-9 hours
- ○ 10-14 hours
- ○ 15-19 hours
- ○ 20+ hours

Please select all that apply in regards to the types of leagues you participated in gymnastics. You may provide your best estimate if you are not exactly sure.

- ☐ I participated in a competitive, school based league
- ☐ I participated in a competitive, non-school based league
- ☐ I participated in a club, recreational, or intramural league at school
- ☐ I participated in a club, recreational, or intramural league outside of school

- [ ] [ ] Other
- [ ] None of the above

In which grades did you participate in ice hockey? Please select all that apply. You may provide your best estimate if you are not exactly sure.

- [ ] Kindergarten
- [ ] Grade 1
- [ ] Grade 2
- [ ] Grade 3
- [ ] Grade 4
- [ ] Grade 5
- [ ] Grade 6

Please select all that apply in regards to your participation in ice hockey. You may provide your best estimate if you are not exactly sure.

- [ ] My team traveled for games or tournaments.
- [ ] My team participated in playoffs.
- [ ] My team won a championship.
- [ ] None of the above

On average, how many hours per week did you spend practicing ice hockey with the team? You may provide your best estimate if you are not exactly sure.

- ○ 1-4 hours

- 5-9 hours
- 10-14 hours
- 15-19 hours
- 20+ hours

On average, how many hours per week did you spend playing in ice hockey games? You may provide your best estimate if you are not exactly sure.

- 1-4 hours
- 5-9 hours
- 10-14 hours
- 15-19 hours
- 20+ hours

Please select all positions you played in. You may provide your best estimate if you are not exactly sure.

- Left/right Defense
- Left/right Wing
- Center
- Goalie
- Other
- None of the above

Please select all that apply in regards to the types of leagues you played ice hockey in. You may provide your best estimate if you are not exactly sure.

- ☐ I participated in a competitive, school based league
- ☐ I participated in a competitive, non-school based league
- ☐ I participated in a club, recreational, or intramural league at school
- ☐ I participated in a club, recreational, or intramural league outside of school
- ☐ ________ Other
- ☐ None of the above

In which grades did you participate in lacrosse? Please select all that apply. You may provide your best estimate if you are not exactly sure.

- ☐ Kindergarten
- ☐ Grade 1
- ☐ Grade 2
- ☐ Grade 3
- ☐ Grade 4
- ☐ Grade 5
- ☐ Grade 6

Please select all that apply in regards to your participation in lacrosse. You may provide your best estimate if you are not exactly sure.

- ☐ My team traveled for games or tournaments.
- ☐ My team participated in playoffs.
- ☐ My team won a championship.
- ☐ None of the above

On average, how many hours per week did you spend practicing lacrosse with the team? You may provide your best estimate if you are not exactly sure.

- ◯ 1-4 hours
- ◯ 5-9 hours
- ◯ 10-14 hours
- ◯ 15-19 hours
- ◯ 20+ hours

On average, how many hours per week did you spend playing in lacrosse games? You may provide your best estimate if you are not exactly sure.

- ◯ 1-4 hours
- ◯ 5-9 hours
- ◯ 10-14 hours
- ◯ 15-19 hours
- ◯ 20+ hours

Please select all positions you played in. You may provide your best estimate if you are not exactly sure.

- ☐ Midfielder
- ☐ Attacker
- ☐ Goalie
- ☐ Defenseman
- ☐ [ ] Other
- ☐ None of the above

Please select all that apply in regards to the types of leagues you played lacrosse in. You may provide your best estimate if you are not exactly sure.

- ☐ I participated in a competitive, school based league
- ☐ I participated in a competitive, non-school based league
- ☐ I participated in a club, recreational, or intramural league at school
- ☐ I participated in a club, recreational, or intramural league outside of school
- ☐ [ ] Other
- ☐ None of the above

In which grades did you participate in rugby? Please select all that apply. You may provide your best estimate if you are not exactly sure.

- ☐ Kindergarten
- ☐ Grade 1
- ☐ Grade 2
- ☐ Grade 3
- ☐ Grade 4
- ☐ Grade 5
- ☐ Grade 6

Please select all that apply in regards to your participation in rugby. You may provide your best estimate if you are not exactly sure.

- ☐ My team traveled for games or tournaments.
- ☐ My team participated in playoffs.
- ☐ My team won a championship.

- ☐ None of the above

On average, how many hours per week did you spend practicing rugby with the team? You may provide your best estimate if you are not exactly sure.

- ○ 1-4 hours
- ○ 5-9 hours
- ○ 10-14 hours
- ○ 15-19 hours
- ○ 20+ hours

On average, how many hours per week did you spend playing in rugby games? You may provide your best estimate if you are not exactly sure.

- ○ 1-4 hours
- ○ 5-9 hours
- ○ 10-14 hours
- ○ 15-19 hours
- ○ 20+ hours

Please select all positions you played in. You may provide your best estimate if you are not exactly sure.

- ☐ Forward
- ☐ Back
- ☐ ________ Other
- ☐ None of the above

Please select all that apply in regards to the types of leagues you played rugby in. You may provide your best estimate if you are not exactly sure.

- ☐ I participated in a competitive, school based league
- ☐ I participated in a competitive, non-school based league
- ☐ I participated in a club, recreational, or intramural league at school
- ☐ I participated in a club, recreational, or intramural league outside of school
- ☐ [ ] Other
- ☐ None of the above

In which grades did you participate in soccer? Please select all that apply. You may provide your best estimate if you are not exactly sure.

- ☐ Kindergarten
- ☐ Grade 1
- ☐ Grade 2
- ☐ Grade 3
- ☐ Grade 4
- ☐ Grade 5
- ☐ Grade 6

Please select all that apply in regards to your participation in soccer. You may provide your best estimate if you are not exactly sure.

- ☐ My team traveled for games or tournaments.
- ☐ My team participated in playoffs.
- ☐ My team won a championship.

☐ None of the above

On average, how many hours per week did you spend practicing soccer with the team? You may provide your best estimate if you are not exactly sure.

○ 1-4 hours
○ 5-9 hours
○ 10-14 hours
○ 15-19 hours
○ 20+ hours

On average, how many hours per week did you spend playing in soccer games? You may provide your best estimate if you are not exactly sure.

○ 1-4 hours
○ 5-9 hours
○ 10-14 hours
○ 15-19 hours
○ 20+ hours

Please select all positions you played in. You may provide your best estimate if you are not exactly sure.

☐ Goalkeeper
☐ Fullback
☐ Center Back
☐ Defending/Holding Midfielder

- ☐ Midfielder/Winger
- ☐ Central/Box-to-Box Midfielder
- ☐ Striker
- ☐ Attacking Midfielder/Playmaker
- ☐ ______ Other
- ☐ None of the above

Please select all that apply in regards to the types of leagues you played soccer in. You may provide your best estimate if you are not exactly sure.

- ☐ I participated in a competitive, school based league
- ☐ I participated in a competitive, non-school based league
- ☐ I participated in a club, recreational, or intramural league at school
- ☐ I participated in a club, recreational, or intramural league outside of school
- ☐ ______ Other
- ☐ None of the above

In which grades did you participate in softball? Please select all that apply. You may provide your best estimate if you are not exactly sure.

- ☐ Kindergarten
- ☐ Grade 1
- ☐ Grade 2
- ☐ Grade 3
- ☐ Grade 4
- ☐ Grade 5
- ☐ Grade 6

Please select all that apply in regards to your participation in softball. You may provide your best estimate if you are not exactly sure.

- ☐ My team traveled for games or tournaments.
- ☐ My team participated in playoffs.
- ☐ My team won a championship.
- ☐ None of the above

On average, how many hours per week did you spend practicing softball with the team? You may provide your best estimate if you are not exactly sure.

- ○ 1-4 hours
- ○ 5-9 hours
- ○ 10-14 hours
- ○ 15-19 hours
- ○ 20+ hours

On average, how many hours per week did you spend playing in softball games? You may provide your best estimate if you are not exactly sure.

- ○ 1-4 hours
- ○ 5-9 hours
- ○ 10-14 hours
- ○ 15-19 hours
- ○ 20+ hours

Please select all positions you played in. You may provide your best estimate if you are not exactly sure.

- ☐ Pitcher
- ☐ Catcher
- ☐ Baseman/Shortstop
- ☐ Outfielder
- ☐ ____ Other
- ☐ None of the above

Please select all that apply in regards to the types of leagues you played softball in. You may provide your best estimate if you are not exactly sure.

- ☐ I participated in a competitive, school based league
- ☐ I participated in a competitive, non-school based league
- ☐ I participated in a club, recreational, or intramural league at school
- ☐ I participated in a club, recreational, or intramural league outside of school
- ☐ ____ Other
- ☐ None of the above

In which grades did you participate in swimming? Please select all that apply. You may provide your best estimate if you are not exactly sure.

- ☐ Kindergarten
- ☐ Grade 1
- ☐ Grade 2
- ☐ Grade 3

- ☐ Grade 4
- ☐ Grade 5
- ☐ Grade 6

Please select all that apply in regards to your participation in swimming. You may provide your best estimate if you are not exactly sure.

- ☐ My team traveled for meets or tournaments.
- ☐ I or my team won a tournament.
- ☐ None of the above

On average, how many hours per week did you spend practicing swimming with the team? You may provide your best estimate if you are not exactly sure.

- ○ 1-4 hours
- ○ 5-9 hours
- ○ 10-14 hours
- ○ 15-19 hours
- ○ 20+ hours

On average, how many hours per week did you spend participating in swim meets? You may provide your best estimate if you are not exactly sure.

- ○ 1-4 hours
- ○ 5-9 hours
- ○ 10-14 hours
- ○ 15-19 hours

○ 20+ hours

Please select all that apply in regards to the types of leagues you participated in swimming. You may provide your best estimate if you are not exactly sure.

☐ I participated in a competitive, school based league
☐ I participated in a competitive, non-school based league
☐ I participated in a club, recreational, or intramural league at school
☐ I participated in a club, recreational, or intramural league outside of school
☐ ________ Other
☐ None of the above

In which grades did you participate in tennis? Please select all that apply. You may provide your best estimate if you are not exactly sure.

☐ Kindergarten
☐ Grade 1
☐ Grade 2
☐ Grade 3
☐ Grade 4
☐ Grade 5
☐ Grade 6

Please select all that apply in regards to your participation in tennis. You may provide your best estimate if you are not exactly sure.

- ☐ My team traveled for meets or tournaments.
- ☐ I or my team won a tournament.
- ☐ None of the above

On average, how many hours per week did you spend practicing tennis with the team? You may provide your best estimate if you are not exactly sure.

- ○ 1-4 hours
- ○ 5-9 hours
- ○ 10-14 hours
- ○ 15-19 hours
- ○ 20+ hours

On average, how many hours per week did you spend participating in tennis matches? You may provide your best estimate if you are not exactly sure.

- ○ 1-4 hours
- ○ 5-9 hours
- ○ 10-14 hours
- ○ 15-19 hours
- ○ 20+ hours

Please select all that apply in regards to the types of leagues you played tennis in. You may provide your best estimate if you are not exactly sure.

- ☐ I participated in a competitive, school based league
- ☐ I participated in a competitive, non-school based league
- ☐ I participated in a club, recreational, or intramural league at school

- [ ] I participated in a club, recreational, or intramural league outside of school
- [ ] Non-school, Travel League
- [ ] ________ Other
- [ ] None of the above

In which grades did you participate in track and field? Please select all that apply. You may provide your best estimate if you are not exactly sure.

- [ ] Kindergarten
- [ ] Grade 1
- [ ] Grade 2
- [ ] Grade 3
- [ ] Grade 4
- [ ] Grade 5
- [ ] Grade 6

Please select all that apply in regards to your participation in track and field. You may provide your best estimate if you are not exactly sure.

- [ ] I or my team traveled for meets or tournaments.
- [ ] I or my team won a tournament.
- [ ] None of the above

On average, how many hours per week did you spend practicing track and field with the team? You may provide your best estimate if you are not exactly sure.

- ○ 1-4 hours
- ○ 5-9 hours
- ○ 10-14 hours
- ○ 15-19 hours
- ○ 20+ hours

On average, how many hours per week did you spend participating in track and field meets? You may provide your best estimate if you are not exactly sure.

- ○ 1-4 hours
- ○ 5-9 hours
- ○ 10-14 hours
- ○ 15-19 hours
- ○ 20+ hours

Please select all that apply in regards to the types of leagues you participated in track and field. You may provide your best estimate if you are not exactly sure.

- ☐ I participated in a competitive, school based league
- ☐ I participated in a competitive, non-school based league
- ☐ I participated in a club, recreational, or intramural league at school
- ☐ I participated in a club, recreational, or intramural league outside of school
- ☐ ________ Other
- ☐ None of the above

In which grades did you participate in volleyball? Please select all that apply. You may provide your best estimate if you are not exactly sure.

- ☐ Kindergarten
- ☐ Grade 1
- ☐ Grade 2
- ☐ Grade 3
- ☐ Grade 4
- ☐ Grade 5
- ☐ Grade 6

Please select all that apply in regards to your participation in volleyball. You may provide your best estimate if you are not exactly sure.

- ☐ My team traveled for games or tournaments.
- ☐ My team participated in playoffs.
- ☐ My team won a championship.
- ☐ None of the above

On average, how many hours per week did you spend practicing volleyball with the team? You may provide your best estimate if you are not exactly sure.

- ○ 1-4 hours
- ○ 5-9 hours
- ○ 10-14 hours
- ○ 15-19 hours
- ○ 20+ hours

On average, how many hours per week did you spend playing in volleyball games? You may provide your best estimate if you are not exactly sure.

- ◯ 1-4 hours
- ◯ 5-9 hours
- ◯ 10-14 hours
- ◯ 15-19 hours
- ◯ 20+ hours

Please select all positions you played in. You may provide your best estimate if you are not exactly sure.

- ☐ Left/right Front
- ☐ Middle Front
- ☐ Left/right Back
- ☐ Middle Back
- ☐ ________ Other
- ☐ None of the above

Please select all that apply in regards to the types of leagues you played volleyball in. You may provide your best estimate if you are not exactly sure.

- ☐ I participated in a competitive, school based league
- ☐ I participated in a competitive, non-school based league
- ☐ I participated in a club, recreational, or intramural league at school
- ☐ I participated in a club, recreational, or intramural league outside of school

- [ ] ______ Other
- [ ] None of the above

In which grades did you participate in water polo? Please select all that apply. You may provide your best estimate if you are not exactly sure.

- [ ] Kindergarten
- [ ] Grade 1
- [ ] Grade 2
- [ ] Grade 3
- [ ] Grade 4
- [ ] Grade 5
- [ ] Grade 6

Please select all that apply in regards to your participation in water polo. You may provide your best estimate if you are not exactly sure.

- [ ] My team traveled for games or tournaments.
- [ ] My team participated in playoffs.
- [ ] My team won a championship.
- [ ] None of the above

On average, how many hours per week did you spend practicing water polo with the team? You may provide your best estimate if you are not exactly sure.

- ( ) 1-4 hours

- 5-9 hours
- 10-14 hours
- 15-19 hours
- 20+ hours

On average, how many hours per week did you spend playing in water polo matches? You may provide your best estimate if you are not exactly sure.

- 1-4 hours
- 5-9 hours
- 10-14 hours
- 15-19 hours
- 20+ hours

Please select all positions you played in. You may provide your best estimate if you are not exactly sure.

- Goalkeeper
- Defensive Specialist
- Driver
- Two-Meter Specialist
- ____________ Other
- None of the above

Please select all that apply in regards to the types of leagues you played water polo in. You may provide your best estimate if you are not exactly

sure.

- ☐ I participated in a competitive, school based league
- ☐ I participated in a competitive, non-school based league
- ☐ I participated in a club, recreational, or intramural league at school
- ☐ I participated in a club, recreational, or intramural league outside of school
- ☐ [ ] Other
- ☐ None of the above

In which grades did you participate in wrestling? Please select all that apply. You may provide your best estimate if you are not exactly sure.

- ☐ Kindergarten
- ☐ Grade 1
- ☐ Grade 2
- ☐ Grade 3
- ☐ Grade 4
- ☐ Grade 5
- ☐ Grade 6

Please select all that apply in regards to your participation in wrestling. You may provide your best estimate if you are not exactly sure.

- ☐ My team traveled for games or tournaments.
- ☐ I or my team won a tournament.
- ☐ None of the above

On average, how many hours per week did you spend practicing wrestling with the team? You may provide your best estimate if you are not exactly sure.

- 1-4 hours
- 5-9 hours
- 10-14 hours
- 15-19 hours
- 20+ hours

On average, how many hours per week did you spend participating in wrestling matches? You may provide your best estimate if you are not exactly sure.

- 1-4 hours
- 5-9 hours
- 10-14 hours
- 15-19 hours
- 20+ hours

Please select all that apply in regards to the types of leagues you participated in wrestling. You may provide your best estimate if you are not exactly sure.

- I participated in a competitive, school based league
- I participated in a competitive, non-school based league
- I participated in a club, recreational, or intramural league at school
- I participated in a club, recreational, or intramural league outside of school
- Other

☐ None of the above

In which grades did you participate in ${q://QID8/ChoiceTextEntryValue/21}? Please select all that apply. You may provide your best estimate if you are not exactly sure.

☐ Kindergarten
☐ Grade 1
☐ Grade 2
☐ Grade 3
☐ Grade 4
☐ Grade 5
☐ Grade 6

Please select all that apply in regards to your participation in ${q://QID8/ChoiceTextEntryValue/21}. You may provide your best estimate if you are not exactly sure.

☐ My team traveled for games or tournaments.
☐ My team participated in playoffs.
☐ My team won a championship.
☐ None of the above

On average, how many hours per week did you spend practicing ${q://QID8/ChoiceTextEntryValue/21} with the team? You may provide your best estimate if you are not exactly sure.

○ 1-4 hours

- 5-9 hours
- 10-14 hours
- 15-19 hours
- 20+ hours

On average, how many hours per week did you spend playing in ${q://QID8/ChoiceTextEntryValue/21} games? You may provide your best estimate if you are not exactly sure.

- 1-4 hours
- 5-9 hours
- 10-14 hours
- 15-19 hours
- 20+ hours

Please indicate the primary position you played while playing ${q://QID8/ChoiceTextEntryValue/21}. You may provide your best estimate if you are not exactly sure.

Please select all that apply in regards to the types of leagues you played ${q://QID8/ChoiceTextEntryValue/21} in. You may provide your best estimate if you are not exactly sure.

- I participated in a competitive, school based league
- I participated in a competitive, non-school based league

- ☐ I participated in a club, recreational, or intramural league at school
- ☐ I participated in a club, recreational, or intramural league outside of school
- ☐ ____________ Other
- ☐ None of the above

In which grades did you participate in ${q://QID8/ChoiceTextEntryValue/22}? Please select all that apply. You may provide your best estimate if you are not exactly sure.

- ☐ Kindergarten
- ☐ Grade 1
- ☐ Grade 2
- ☐ Grade 3
- ☐ Grade 4
- ☐ Grade 5
- ☐ Grade 6

Please select all that apply in regards to your participation in ${q://QID8/ChoiceTextEntryValue/22}. You may provide your best estimate if you are not exactly sure.

- ☐ My team traveled for games or tournaments.
- ☐ My team participated in playoffs.
- ☐ My team won a championship.
- ☐ None of the above

On average, how many hours per week did you spend practicing ${q://QID8/ChoiceTextEntryValue/22} with the team? You may provide your best estimate if you are not exactly sure.

- 1-4 hours
- 5-9 hours
- 10-14 hours
- 15-19 hours
- 20+ hours

On average, how many hours per week did you spend playing in ${q://QID8/ChoiceTextEntryValue/22} games?

- 1-4 hours
- 5-9 hours
- 10-14 hours
- 15-19 hours
- 20+ hours

Please indicate the primary position you played while playing ${q://QID8/ChoiceTextEntryValue/22}. You may provide your best estimate if you are not exactly sure.

Please select all that apply in regards to the types of leagues you played ${q://QID8/ChoiceTextEntryValue/22} in. You may provide your best

estimate if you are not exactly sure.

- ☐ I participated in a competitive, school based league
- ☐ I participated in a competitive, non-school based league
- ☐ I participated in a club, recreational, or intramural league at school
- ☐ I participated in a club, recreational, or intramural league outside of school
- ☐ [ ] Other
- ☐ None of the above

In which grades did you participate in ${q://QID8/ChoiceTextEntryValue/23}? Please select all that apply. You may provide your best estimate if you are not exactly sure.

- ☐ Kindergarten
- ☐ Grade 1
- ☐ Grade 2
- ☐ Grade 3
- ☐ Grade 4
- ☐ Grade 5
- ☐ Grade 6

Please select all that apply in regards to your participation in ${q://QID8/ChoiceTextEntryValue/23}. You may provide your best estimate if you are not exactly sure.

- ☐ My team traveled for games or tournaments.
- ☐ My team participated in playoffs.
- ☐ My team won a championship.
- ☐ None of the above

On average, how many hours per week did you spend practicing ${q://QID8/ChoiceTextEntryValue/23} with the team? You may provide your best estimate if you are not exactly sure.

- 1-4 hours
- 5-9 hours
- 10-14 hours
- 15-19 hours
- 20+ hours

On average, how many hours per week did you spend playing in ${q://QID8/ChoiceTextEntryValue/23} games? You may provide your best estimate if you are not exactly sure.

- 1-4 hours
- 5-9 hours
- 10-14 hours
- 15-19 hours
- 20+ hours

Please indicate the primary position you played while playing ${q://QID8/ChoiceTextEntryValue/23}. You may provide your best estimate if you are not exactly sure.

Please select all that apply in regards to the types of leagues you played ${q://QID8/ChoiceTextEntryValue/23} in. You may provide your best estimate if you are not exactly sure.

- ☐ I participated in a competitive, school based league
- ☐ I participated in a competitive, non-school based league
- ☐ I participated in a club, recreational, or intramural league at school
- ☐ I participated in a club, recreational, or intramural league outside of school
- ☐ [ ] Other
- ☐ None of the above

Please detail your participation in any other regular athletic participation outside of an organized team from Kindergarten through Grade 6.

[ ]

Grades 7-8

Please select all sports that you participated in on an organized team at any time during Grade 7 or Grade 8.

If a sport you participated in is not listed, you may select "Other Sport" and type in up to three other sports.

If you did not participate in any sports, please select "None."

- ☐ None
- ☐ American Flag Football
- ☐ American Tackle Football
- ☐ Baseball
- ☐ Basketball
- ☐ Cheerleading
- ☐ Cross Country
- ☐ Field Hockey
- ☐ Golf
- ☐ Gymnastics
- ☐ Ice Hockey
- ☐ Lacrosse
- ☐ Rugby
- ☐ Soccer
- ☐ Softball
- ☐ Swimming
- ☐ Tennis
- ☐ Track & Field
- ☐ Volleyball
- ☐ Water Polo
- ☐ Wrestling
- ☐ ______ Other Sport 1
- ☐ ______ Other Sport 2
- ☐ ______ Other Sport 3

Did you have any physical limitations that prevented you from playing on an organized sports team in Grade 7 and/or Grade 8?

- Yes
- No
- I prefer not to answer

Note. The following questions are in regards to your participation in sports in Grade 7 and Grade 8.

In which grades did you play baseball? Please select all that apply. You may provide your best estimate if you are not exactly sure.

- [ ] Grade 7
- [ ] Grade 8

Please select all that apply in regards to your participation in baseball. You may provide your best estimate if you are not exactly sure.

- [ ] My team traveled for games or tournaments.
- [ ] My team participated in playoffs.
- [ ] My team won a championship.
- [ ] None of the above

On average, how many hours per week did you spend practicing baseball with the team? You may provide your best estimate if you are not exactly sure.

- ○ 1-4 hours
- ○ 5-9 hours
- ○ 10-14 hours
- ○ 15-19 hours
- ○ 20+ hours

On average, how many hours per week did you spend playing in baseball games? You may provide your best estimate if you are not exactly sure.

- ○ 1-4 hours
- ○ 5-9 hours
- ○ 10-14 hours
- ○ 15-19 hours
- ○ 20+ hours

Please select all positions you played in. You may provide your best estimate if you are not exactly sure.

- ☐ Pitcher
- ☐ Catcher
- ☐ Baseman/Shortstop
- ☐ Outfielder
- ☐ [ ] Other
- ☐ None of the above

Please select all that apply in regards to the types of leagues you played baseball in. You may provide your best estimate if you are not exactly sure.

- ☐ I participated in a competitive, school based league
- ☐ I participated in a competitive, non-school based league
- ☐ I participated in a club, recreational, or intramural league at school
- ☐ I participated in a club, recreational, or intramural league outside of school
- ☐ ______ Other
- ☐ None of the above

In which grades did you play basketball? Please select all that apply. You may provide your best estimate if you are not exactly sure.

- ☐ Grade 7
- ☐ Grade 8

Please select all that apply in regards to your participation in basketball. You may provide your best estimate if you are not exactly sure.

- ☐ My team traveled for games or tournaments.
- ☐ My team participated in playoffs.
- ☐ My team won a championship.
- ☐ None of the above

On average, how many hours per week did you spend practicing basketball with the team? You may provide your best estimate if you are not exactly sure.

- 1-4 hours
- 5-9 hours
- 10-14 hours
- 15-19 hours
- 20+ hours

On average, how many hours per week did you spend playing in basketball games? You may provide your best estimate if you are not exactly sure.

- 1-4 hours
- 5-9 hours
- 10-14 hours
- 15-19 hours
- 20+ hours

Please select all positions you played in. You may provide your best estimate if you are not exactly sure.

- Guard
- Forward
- Center
- Other
- None of the above

Please select all that apply in regards to the types of leagues you played basketball in. You may provide your best estimate if you are not exactly sure.

- ☐ I participated in a competitive, school based league
- ☐ I participated in a competitive, non-school based league
- ☐ I participated in a club, recreational, or intramural league at school
- ☐ I participated in a club, recreational, or intramural league outside of school
- ☐ [ ] Other
- ☐ None of the above

In which grades did you participate in cheerleading? Please select all that apply. You may provide your best estimate if you are not exactly sure.

- ☐ Grade 7
- ☐ Grade 8

Please select all that apply in regards to your participation in cheerleading. You may provide your best estimate if you are not exactly sure.

- ☐ I traveled for cheerleading tournaments.
- ☐ My team won a cheerleading tournament.
- ☐ None of the above

On average, how many hours per week did you spend practicing cheerleading? You may provide your best estimate if you are not exactly sure.

- 1-4 hours
- 5-9 hours
- 10-14 hours
- 15-19 hours
- 20+ hours

Please select all that apply in regards to the types of leagues you participated in cheerleading. You may provide your best estimate if you are not exactly sure.

- I participated in a competitive, school based league
- I participated in a competitive, non-school based league
- I participated in a club, recreational, or intramural league at school
- I participated in a club, recreational, or intramural league outside of school
- Other
- None of the above

In which grades did you participate in cross country? Please select all that apply. You may provide your best estimate if you are not exactly sure.

- Grade 7
- Grade 8

Please select all that apply in regards to your participation in cross country. You may provide your best estimate if you are not exactly sure.

- ☐ I traveled for meets or tournaments.
- ☐ I or my team won a tournament.
- ☐ None of the above

On average, how many hours per week did you spend practicing cross country with the team? You may provide your best estimate if you are not exactly sure.

- ○ 1-4 hours
- ○ 5-9 hours
- ○ 10-14 hours
- ○ 15-19 hours
- ○ 20+ hours

On average, how many hours per week did you spend participating in cross country meets? You may provide your best estimate if you are not exactly sure.

- ○ 1-4 hours
- ○ 5-9 hours
- ○ 10-14 hours
- ○ 15-19 hours
- ○ 20+ hours

Please select all that apply in regards to the types of leagues you participated in cross country. You may provide your best estimate if you are not exactly sure.

- ☐ I participated in a competitive, school based league
- ☐ I participated in a competitive, non-school based league
- ☐ I participated in a club, recreational, or intramural league at school
- ☐ I participated in a club, recreational, or intramural league outside of school
- ☐ Other
- ☐ None of the above

In which grades did you participate in field hockey? Please select all that apply. You may provide your best estimate if you are not exactly sure.

- ☐ Grade 7
- ☐ Grade 8

Please select all that apply in regards to your participation in field hockey. You may provide your best estimate if you are not exactly sure.

- ☐ My team traveled for games or tournaments.
- ☐ My team participated in playoffs.
- ☐ My team won a championship.
- ☐ None of the above

On average, how many hours per week did you spend practicing field hockey with the team? You may provide your best estimate if you are not

exactly sure.

- ◯ 1-4 hours
- ◯ 5-9 hours
- ◯ 10-14 hours
- ◯ 15-19 hours
- ◯ 20+ hours

On average, how many hours per week did you spend playing in field hockey games? You may provide your best estimate if you are not exactly sure.

- ◯ 1-4 hours
- ◯ 5-9 hours
- ◯ 10-14 hours
- ◯ 15-19 hours
- ◯ 20+ hours

Please select all positions you played in. You may provide your best estimate if you are not exactly sure.

- ☐ Left/right Defense
- ☐ Left/right Wing
- ☐ Center
- ☐ Goalie
- ☐ ____________ Other
- ☐ None of the above

Please select all that apply in regards to the types of leagues you played field hockey in. You may provide your best estimate if you are not exactly sure.

- ☐ I participated in a competitive, school based league
- ☐ I participated in a competitive, non-school based league
- ☐ I participated in a club, recreational, or intramural league at school
- ☐ I participated in a club, recreational, or intramural league outside of school
- ☐ ________ Other
- ☐ None of the above

In which grades did you participate in American tackle football? Please select all that apply. You may provide your best estimate if you are not exactly sure.

- ☐ Grade 7
- ☐ Grade 8

Please select all that apply in regards to your participation in American tackle football. You may provide your best estimate if you are not exactly sure.

- ☐ My team traveled for games or tournaments.
- ☐ My team participated in playoffs.
- ☐ My team won a championship.
- ☐ None of the above

On average, how many hours per week did you spend practicing American tackle football with the team? You may provide your best estimate if you are not exactly sure.

- ◯ 1-4 hours
- ◯ 5-9 hours
- ◯ 10-14 hours
- ◯ 15-19 hours
- ◯ 20+ hours

On average, how many hours per week did you spend playing in American tackle football games? You may provide your best estimate if you are not exactly sure.

- ◯ 1-4 hours
- ◯ 5-9 hours
- ◯ 10-14 hours
- ◯ 15-19 hours
- ◯ 20+ hours

Please select all positions you played in. You may provide your best estimate if you are not exactly sure.

- ☐ Quarterback
- ☐ Running Back
- ☐ Fullback
- ☐ Offensive Line
- ☐ Wide Receiver
- ☐ Tight End

- ☐ Defensive Line
- ☐ Linebacker
- ☐ Cornerback
- ☐ Safety
- ☐ Kicker/Punter
- ☐ Participation in kick off return
- ☐ Long Snapper
- ☐ ________ Other
- ☐ None of the above

Please select all that apply in regards to the types of leagues you played American tackle football in. You may provide your best estimate if you are not exactly sure.

- ☐ I participated in a competitive, school based league
- ☐ I participated in a competitive, non-school based league
- ☐ I participated in a club, recreational, or intramural league at school
- ☐ I participated in a club, recreational, or intramural league outside of school
- ☐ ________ Other
- ☐ None of the above

In which grades did you participate in American flag football? Please select all that apply. You may provide your best estimate if you are not exactly sure.

- ☐ Grade 7
- ☐ Grade 8

Please select all that apply in regards to your participation in American flag football. You may provide your best estimate if you are not exactly sure.

- ☐ My team traveled for games or tournaments.
- ☐ My team participated in playoffs.
- ☐ My team won a championship.
- ☐ None of the above

On average, how many hours per week did you spend practicing American flag football with the team? You may provide your best estimate if you are not exactly sure.

- ◯ 1-4 hours
- ◯ 5-9 hours
- ◯ 10-14 hours
- ◯ 15-19 hours
- ◯ 20+ hours

On average, how many hours per week did you spend playing in American flag football games? You may provide your best estimate if you are not exactly sure.

- ◯ 1-4 hours
- ◯ 5-9 hours
- ◯ 10-14 hours
- ◯ 15-19 hours
- ◯ 20+ hours

Please select all positions you played in. You may provide your best estimate if you are not exactly sure.

- ☐ Quarterback
- ☐ Running Back
- ☐ Fullback
- ☐ Offensive Line
- ☐ Wide Receiver
- ☐ Tight End
- ☐ Defensive Line
- ☐ Linebacker
- ☐ Cornerback
- ☐ Safety
- ☐ Kicker/Punter
- ☐ Participation in kick off return
- ☐ Long Snapper
- ☐ [ ] Other
- ☐ None of the above

Please select all that apply in regards to the types of leagues you played American flag football in. You may provide your best estimate if you are not exactly sure.

- ☐ I participated in a competitive, school based league
- ☐ I participated in a competitive, non-school based league
- ☐ I participated in a club, recreational, or intramural league at school
- ☐ I participated in a club, recreational, or intramural league outside of school
- ☐ [ ] Other

☐ None of the above

In which grades did you participate in golf? Please select all that apply. You may provide your best estimate if you are not exactly sure.

☐ Grade 7
☐ Grade 8

Please select all that apply in regards to your participation in golf. You may provide your best estimate if you are not exactly sure.

☐ My team traveled for matches or tournaments.
☐ I or my team won a tournament.
☐ None of the above

On average, how many hours per week did you spend practicing golf with the team? You may provide your best estimate if you are not exactly sure.

○ 1-4 hours
○ 5-9 hours
○ 10-14 hours
○ 15-19 hours
○ 20+ hours

On average, how many hours per week did you spend participating in golf matches? You may provide your best estimate if you are not exactly sure.

- 1-4 hours
- 5-9 hours
- 10-14 hours
- 15-19 hours
- 20+ hours

Please select all that apply in regards to the types of leagues you participated in golf. You may provide your best estimate if you are not exactly sure.

- I participated in a competitive, school based league
- I participated in a competitive, non-school based league
- I participated in a club, recreational, or intramural league at school
- I participated in a club, recreational, or intramural league outside of school
- Other
- None of the above

In which grades did you participate in gymnastics? Please select all that apply. You may provide your best estimate if you are not exactly sure.

- Grade 7
- Grade 8

Please select all that apply in regards to your participation in gymnastics. You may provide your best estimate if you are not exactly sure.

- My team traveled for meets or tournaments.

- [ ] I or my team won a tournament.
- [ ] None of the above

On average, how many hours per week did you spend practicing gymnastics with the team? You may provide your best estimate if you are not exactly sure.

- ○ 1-4 hours
- ○ 5-9 hours
- ○ 10-14 hours
- ○ 15-19 hours
- ○ 20+ hours

On average, how many hours per week did you spend participating in gymnastics meets? You may provide your best estimate if you are not exactly sure.

- ○ 1-4 hours
- ○ 5-9 hours
- ○ 10-14 hours
- ○ 15-19 hours
- ○ 20+ hours

Please select all that apply in regards to the types of leagues you participated in gymnastics. You may provide your best estimate if you are not exactly sure.

- [ ] I participated in a competitive, school based league

- ☐ I participated in a competitive, non-school based league
- ☐ I participated in a club, recreational, or intramural league at school
- ☐ I participated in a club, recreational, or intramural league outside of school
- ☐ [ ] Other
- ☐ None of the above

In which grades did you participate in ice hockey? Please select all that apply. You may provide your best estimate if you are not exactly sure.

- ☐ Grade 7
- ☐ Grade 8

Please select all that apply in regards to your participation in ice hockey. You may provide your best estimate if you are not exactly sure.

- ☐ My team traveled for games or tournaments.
- ☐ My team participated in playoffs.
- ☐ My team won a championship.
- ☐ None of the above

On average, how many hours per week did you spend practicing ice hockey with the team? You may provide your best estimate if you are not exactly sure.

- ○ 1-4 hours
- ○ 5-9 hours
- ○ 10-14 hours

- ○ 15-19 hours
- ○ 20+ hours

On average, how many hours per week did you spend playing in ice hockey games? You may provide your best estimate if you are not exactly sure.

- ○ 1-4 hours
- ○ 5-9 hours
- ○ 10-14 hours
- ○ 15-19 hours
- ○ 20+ hours

Please select all positions you played in. You may provide your best estimate if you are not exactly sure.

- ☐ Left/right Defense
- ☐ Left/right Wing
- ☐ Center
- ☐ Goalie
- ☐ [ ] Other
- ☐ None of the above

Please select all that apply in regards to the types of leagues you played ice hockey in. You may provide your best estimate if you are not exactly sure.

- ☐ I participated in a competitive, school based league

- ☐ I participated in a competitive, non-school based league
- ☐ I participated in a club, recreational, or intramural league at school
- ☐ I participated in a club, recreational, or intramural league outside of school
- ☐ [ ] Other
- ☐ None of the above

In which grades did you participate in lacrosse? Please select all that apply. You may provide your best estimate if you are not exactly sure.

- ☐ Grade 7
- ☐ Grade 8

Please select all that apply in regards to your participation in lacrosse. You may provide your best estimate if you are not exactly sure.

- ☐ My team traveled for games or tournaments.
- ☐ My team participated in playoffs.
- ☐ My team won a championship.
- ☐ None of the above

On average, how many hours per week did you spend practicing lacrosse with the team? You may provide your best estimate if you are not exactly sure.

- ○ 1-4 hours
- ○ 5-9 hours
- ○ 10-14 hours

- 15-19 hours
- 20+ hours

On average, how many hours per week did you spend playing in lacrosse games? You may provide your best estimate if you are not exactly sure.

- 1-4 hours
- 5-9 hours
- 10-14 hours
- 15-19 hours
- 20+ hours

Please select all positions you played in. You may provide your best estimate if you are not exactly sure.

- Midfielder
- Attacker
- Goalie
- Defenseman
- Other
- None of the above

Please select all that apply in regards to the types of leagues you played lacrosse in. You may provide your best estimate if you are not exactly sure.

- I participated in a competitive, school based league
- I participated in a competitive, non-school based league

- ☐ I participated in a club, recreational, or intramural league at school
- ☐ I participated in a club, recreational, or intramural league outside of school
- ☐ ________ Other
- ☐ None of the above

In which grades did you participate in rugby? Please select all that apply. You may provide your best estimate if you are not exactly sure.

- ☐ Grade 7
- ☐ Grade 8

Please select all that apply in regards to your participation in rugby. You may provide your best estimate if you are not exactly sure.

- ☐ My team traveled for games or tournaments.
- ☐ My team participated in playoffs.
- ☐ My team won a championship.
- ☐ None of the above

On average, how many hours per week did you spend practicing rugby with the team? You may provide your best estimate if you are not exactly sure.

- ○ 1-4 hours
- ○ 5-9 hours
- ○ 10-14 hours
- ○ 15-19 hours
- ○ 20+ hours

On average, how many hours per week did you spend playing in rugby games? You may provide your best estimate if you are not exactly sure.

- 1-4 hours
- 5-9 hours
- 10-14 hours
- 15-19 hours
- 20+ hours

Please select all positions you played in. You may provide your best estimate if you are not exactly sure.

- Forward
- Back
- Other
- None of the above

Please select all that apply in regards to the types of leagues you played rugby in. You may provide your best estimate if you are not exactly sure.

- I participated in a competitive, school based league
- I participated in a competitive, non-school based league
- I participated in a club, recreational, or intramural league at school
- I participated in a club, recreational, or intramural league outside of school
- Other
- None of the above

In which grades did you participate in soccer? Please select all that apply. You may provide your best estimate if you are not exactly sure.

- ☐ Grade 7
- ☐ Grade 8

Please select all that apply in regards to your participation in soccer. You may provide your best estimate if you are not exactly sure.

- ☐ My team traveled for games or tournaments.
- ☐ My team participated in playoffs.
- ☐ My team won a championship.
- ☐ None of the above

On average, how many hours per week did you spend practicing soccer with the team? You may provide your best estimate if you are not exactly sure.

- ○ 1-4 hours
- ○ 5-9 hours
- ○ 10-14 hours
- ○ 15-19 hours
- ○ 20+ hours

On average, how many hours per week did you spend playing in soccer games? You may provide your best estimate if you are not exactly sure.

- ◯ 1-4 hours
- ◯ 5-9 hours
- ◯ 10-14 hours
- ◯ 15-19 hours
- ◯ 20+ hours

Please select all positions you played in. You may provide your best estimate if you are not exactly sure.

- ☐ Goalkeeper
- ☐ Fullback
- ☐ Center Back
- ☐ Defending/Holding Midfielder
- ☐ Midfielder/Winger
- ☐ Central/Box-to-Box Midfielder
- ☐ Striker
- ☐ Attacking Midfielder/Playmaker
- ☐ ________ Other
- ☐ None of the above

Please select all that apply in regards to the types of leagues you played soccer in. You may provide your best estimate if you are not exactly sure.

- ☐ I participated in a competitive, school based league
- ☐ I participated in a competitive, non-school based league

- ☐ I participated in a club, recreational, or intramural league at school
- ☐ I participated in a club, recreational, or intramural league outside of school
- ☐ ________ Other
- ☐ None of the above

In which grades did you participate in softball? Please select all that apply. You may provide your best estimate if you are not exactly sure.

- ☐ Grade 7
- ☐ Grade 8

Please select all that apply in regards to your participation in softball. You may provide your best estimate if you are not exactly sure.

- ☐ My team traveled for games or tournaments.
- ☐ My team participated in playoffs.
- ☐ My team won a championship.
- ☐ None of the above

On average, how many hours per week did you spend practicing softball with the team? You may provide your best estimate if you are not exactly sure.

- ○ 1-4 hours
- ○ 5-9 hours
- ○ 10-14 hours
- ○ 15-19 hours

- ○ 20+ hours

On average, how many hours per week did you spend playing in softball games? You may provide your best estimate if you are not exactly sure.

- ○ 1-4 hours
- ○ 5-9 hours
- ○ 10-14 hours
- ○ 15-19 hours
- ○ 20+ hours

Please select all positions you played in. You may provide your best estimate if you are not exactly sure.

- ☐ Pitcher
- ☐ Catcher
- ☐ Baseman/Shortstop
- ☐ Outfielder
- ☐ ________ Other
- ☐ None of the above

Please select all that apply in regards to the types of leagues you played softball in. You may provide your best estimate if you are not exactly sure.

- ☐ I participated in a competitive, school based league
- ☐ I participated in a competitive, non-school based league
- ☐ I participated in a club, recreational, or intramural league at school

- ☐ I participated in a club, recreational, or intramural league outside of school
- ☐ [ ] Other
- ☐ None of the above

In which grades did you participate in swimming? Please select all that apply. You may provide your best estimate if you are not exactly sure.

- ☐ Grade 7
- ☐ Grade 8

Please select all that apply in regards to your participation in swimming. You may provide your best estimate if you are not exactly sure.

- ☐ My team traveled for meets or tournaments.
- ☐ I or my team won a tournament.
- ☐ None of the above

On average, how many hours per week did you spend practicing swimming with the team? You may provide your best estimate if you are not exactly sure.

- ○ 1-4 hours
- ○ 5-9 hours
- ○ 10-14 hours
- ○ 15-19 hours
- ○ 20+ hours

On average, how many hours per week did you spend participating in swim meets? You may provide your best estimate if you are not exactly sure.

- ◯ 1-4 hours
- ◯ 5-9 hours
- ◯ 10-14 hours
- ◯ 15-19 hours
- ◯ 20+ hours

Please select all that apply in regards to the types of leagues you participated in swimming. You may provide your best estimate if you are not exactly sure.

- ☐ I participated in a competitive, school based league
- ☐ I participated in a competitive, non-school based league
- ☐ I participated in a club, recreational, or intramural league at school
- ☐ I participated in a club, recreational, or intramural league outside of school
- ☐ [ ] Other
- ☐ None of the above

In which grades did you participate in tennis? Please select all that apply. You may provide your best estimate if you are not exactly sure.

- ☐ Grade 7
- ☐ Grade 8

Please select all that apply in regards to your participation in tennis. You may provide your best estimate if you are not exactly sure.

- ☐ My team traveled for meets or tournaments.
- ☐ I or my team won a tournament.
- ☐ None of the above

On average, how many hours per week did you spend practicing tennis with the team? You may provide your best estimate if you are not exactly sure.

- ○ 1-4 hours
- ○ 5-9 hours
- ○ 10-14 hours
- ○ 15-19 hours
- ○ 20+ hours

On average, how many hours per week did you spend participating in tennis matches? You may provide your best estimate if you are not exactly sure.

- ○ 1-4 hours
- ○ 5-9 hours
- ○ 10-14 hours
- ○ 15-19 hours
- ○ 20+ hours

Please select all that apply in regards to the types of leagues you played tennis in. You may provide your best estimate if you are not exactly sure.

- ○ I participated in a competitive, school based league
- ○ I participated in a competitive, non-school based league
- ○ I participated in a club, recreational, or intramural league at school
- ○ I participated in a club, recreational, or intramural league outside of school
- ○ [ ] Other
- ○ None of the above

In which grades did you participate in track and field? Please select all that apply. You may provide your best estimate if you are not exactly sure.

- ☐ Grade 7
- ☐ Grade 8

Please select all that apply in regards to your participation in track and field. You may provide your best estimate if you are not exactly sure.

- ☐ I or my team traveled for meets or tournaments.
- ☐ I or my team won a tournament.
- ☐ None of the above

On average, how many hours per week did you spend practicing track and field with the team? You may provide your best estimate if you are not exactly sure.

- ○ 1-4 hours
- ○ 5-9 hours
- ○ 10-14 hours

- ◯ 15-19 hours
- ◯ 20+ hours

On average, how many hours per week did you spend participating in track and field meets? You may provide your best estimate if you are not exactly sure.

- ◯ 1-4 hours
- ◯ 5-9 hours
- ◯ 10-14 hours
- ◯ 15-19 hours
- ◯ 20+ hours

Please select all that apply in regards to the types of leagues you participated in track and field. You may provide your best estimate if you are not exactly sure.

- ☐ I participated in a competitive, school based league
- ☐ I participated in a competitive, non-school based league
- ☐ I participated in a club, recreational, or intramural league at school
- ☐ I participated in a club, recreational, or intramural league outside of school
- ☐ ________ Other
- ☐ None of the above

In which grades did you participate in volleyball? Please select all that apply. You may provide your best estimate if you are not exactly sure.

- ☐ Grade 7
- ☐ Grade 8

Please select all that apply in regards to your participation in volleyball. You may provide your best estimate if you are not exactly sure.

- ☐ My team traveled for games or tournaments.
- ☐ My team participated in playoffs.
- ☐ My team won a championship.
- ☐ None of the above

On average, how many hours per week did you spend practicing volleyball with the team? You may provide your best estimate if you are not exactly sure.

- ○ 1-4 hours
- ○ 5-9 hours
- ○ 10-14 hours
- ○ 15-19 hours
- ○ 20+ hours

On average, how many hours per week did you spend playing in volleyball games? You may provide your best estimate if you are not exactly sure.

- ○ 1-4 hours
- ○ 5-9 hours
- ○ 10-14 hours

○ 15-19 hours
○ 20+ hours

Please select all positions you played in. You may provide your best estimate if you are not exactly sure.

☐ Left/right Front
☐ Middle Front
☐ Left/right Back
☐ Middle Back
☐ [ ] Other
☐ None of the above

Please select all that apply in regards to the types of leagues you played volleyball in. You may provide your best estimate if you are not exactly sure.

☐ I participated in a competitive, school based league
☐ I participated in a competitive, non-school based league
☐ I participated in a club, recreational, or intramural league at school
☐ I participated in a club, recreational, or intramural league outside of school
☐ [ ] Other
☐ None of the above

In which grades did you participate in water polo? Please select all that apply. You may provide your best estimate if you are not exactly sure.

☐ Grade 7
☐ Grade 8

Please select all that apply in regards to your participation in water polo. You may provide your best estimate if you are not exactly sure.

☐ My team traveled for games or tournaments.
☐ My team participated in playoffs.
☐ My team won a championship.
☐ None of the above

On average, how many hours per week did you spend practicing water polo with the team? You may provide your best estimate if you are not exactly sure.

○ 1-4 hours
○ 5-9 hours
○ 10-14 hours
○ 15-19 hours
○ 20+ hours

On average, how many hours per week did you spend playing in water polo matches? You may provide your best estimate if you are not exactly sure.

○ 1-4 hours
○ 5-9 hours
○ 10-14 hours

- ○ 15-19 hours
- ○ 20+ hours

Please select all positions you played in. You may provide your best estimate if you are not exactly sure.

- ☐ Goalkeeper
- ☐ Defensive Specialist
- ☐ Driver
- ☐ Two-Meter Specialist
- ☐ [ ] Other
- ☐ None of the above

Please select all that apply in regards to the types of leagues you played water polo in. You may provide your best estimate if you are not exactly sure.

- ☐ I participated in a competitive, school based league
- ☐ I participated in a competitive, non-school based league
- ☐ I participated in a club, recreational, or intramural league at school
- ☐ I participated in a club, recreational, or intramural league outside of school
- ☐ [ ] Other
- ☐ None of the above

In which grades did you participate in wrestling? Please select all that apply. You may provide your best estimate if you are not exactly sure.

- ☐ Grade 7
- ☐ Grade 8

Please select all that apply in regards to your participation in wrestling. You may provide your best estimate if you are not exactly sure.

- ☐ My team traveled for games or tournaments.
- ☐ I or my team won a tournament.
- ☐ None of the above

On average, how many hours per week did you spend practicing wrestling with the team? You may provide your best estimate if you are not exactly sure.

- ○ 1-4 hours
- ○ 5-9 hours
- ○ 10-14 hours
- ○ 15-19 hours
- ○ 20+ hours

On average, how many hours per week did you spend participating in wrestling matches? You may provide your best estimate if you are not exactly sure.

- ○ 1-4 hours
- ○ 5-9 hours
- ○ 10-14 hours
- ○ 15-19 hours

- 20+ hours

Please select all that apply in regards to the types of leagues you participated in wrestling. You may provide your best estimate if you are not exactly sure.

- I participated in a competitive, school based league
- I participated in a competitive, non-school based league
- I participated in a club, recreational, or intramural league at school
- I participated in a club, recreational, or intramural league outside of school
- Other
- None of the above

In which grades did you participate in ${q://QID113/ChoiceTextEntryValue/21}? Please select all that apply. You may provide your best estimate if you are not exactly sure.

- Grade 7
- Grade 8

Please select all that apply in regards to your participation in ${q://QID113/ChoiceTextEntryValue/21}. You may provide your best estimate if you are not exactly sure.

- My team traveled for games or tournaments.
- My team participated in playoffs.
- My team won a championship.

☐ None of the above

On average, how many hours per week did you spend practicing ${q://QID113/ChoiceTextEntryValue/21} with the team? You may provide your best estimate if you are not exactly sure.

◯ 1-4 hours
◯ 5-9 hours
◯ 10-14 hours
◯ 15-19 hours
◯ 20+ hours

On average, how many hours per week did you spend playing in ${q://QID113/ChoiceTextEntryValue/21} games? You may provide your best estimate if you are not exactly sure.

◯ 1-4 hours
◯ 5-9 hours
◯ 10-14 hours
◯ 15-19 hours
◯ 20+ hours

Please indicate the primary position you played while playing ${q://QID113/ChoiceTextEntryValue/21}. You may provide your best estimate if you are not exactly sure.

Please select all that apply in regards to the types of leagues you played ${q://QID113/ChoiceTextEntryValue/21} in. You may provide your best estimate if you are not exactly sure.

- ☐ I participated in a competitive, school based league
- ☐ I participated in a competitive, non-school based league
- ☐ I participated in a club, recreational, or intramural league at school
- ☐ I participated in a club, recreational, or intramural league outside of school
- ☐ ________ Other
- ☐ None of the above

In which grades did you participate in ${q://QID113/ChoiceTextEntryValue/22}? Please select all that apply. You may provide your best estimate if you are not exactly sure.

- ☐ Grade 7
- ☐ Grade 8

Please select all that apply in regards to your participation in ${q://QID113/ChoiceTextEntryValue/22}. You may provide your best estimate if you are not exactly sure.

- ☐ My team traveled for games or tournaments.
- ☐ My team participated in playoffs.
- ☐ My team won a championship.
- ☐ None of the above

On average, how many hours per week did you spend practicing ${q://QID113/ChoiceTextEntryValue/22} with the team? You may provide your best estimate if you are not exactly sure.

- 1-4 hours
- 5-9 hours
- 10-14 hours
- 15-19 hours
- 20+ hours

On average, how many hours per week did you spend playing in ${q://QID113/ChoiceTextEntryValue/22} games? You may provide your best estimate if you are not exactly sure.

- 1-4 hours
- 5-9 hours
- 10-14 hours
- 15-19 hours
- 20+ hours

Please indicate the primary position you played while playing ${q://QID113/ChoiceTextEntryValue/22}. You may provide your best estimate if you are not exactly sure.

Please select all that apply in regards to the types of leagues you played ${q://QID113/ChoiceTextEntryValue/22} in. You may provide your best estimate if you are not exactly sure.

- ☐ I participated in a competitive, school based league
- ☐ I participated in a competitive, non-school based league
- ☐ I participated in a club, recreational, or intramural league at school
- ☐ I participated in a club, recreational, or intramural league outside of school
- ☐ [ ] Other
- ☐ None of the above

In which grades did you participate in ${q://QID113/ChoiceTextEntryValue/23}? Please select all that apply. You may provide your best estimate if you are not exactly sure.

- ☐ Grade 7
- ☐ Grade 8

Please select all that apply in regards to your participation in ${q://QID113/ChoiceTextEntryValue/23}. You may provide your best estimate if you are not exactly sure.

- ☐ My team traveled for games or tournaments.
- ☐ My team participated in playoffs.
- ☐ My team won a championship.
- ☐ None of the above

On average, how many hours per week did you spend practicing ${q://QID113/ChoiceTextEntryValue/23} with the team? You may provide your best estimate if you are not exactly sure.

- 1-4 hours
- 5-9 hours
- 10-14 hours
- 15-19 hours
- 20+ hours

On average, how many hours per week did you spend playing in ${q://QID113/ChoiceTextEntryValue/23} games? You may provide your best estimate if you are not exactly sure.

- 1-4 hours
- 5-9 hours
- 10-14 hours
- 15-19 hours
- 20+ hours

Please indicate the primary position you played while playing ${q://QID113/ChoiceTextEntryValue/23}.

Please select all that apply in regards to the types of leagues you played ${q://QID113/ChoiceTextEntryValue/23} in. You may provide your

best estimate if you are not exactly sure.

- ☐ I participated in a competitive, school based league
- ☐ I participated in a competitive, non-school based league
- ☐ I participated in a club, recreational, or intramural league at school
- ☐ I participated in a club, recreational, or intramural league outside of school
- ☐ [ ] Other
- ☐ None of the above

Please detail your participation in any other regular athletic participation outside of an organized team in Grade 7 and/or Grade 8.

Grades 9-12

Please select all sports that you participated in on an organized team at any time during High School (Grade 9 through Grade 12).

If a sport you participated in is not listed, you may select "Other Sport" and type in up to three other sports.

If you did not participate in any sports, please select "None."

- ☐ None
- ☐ American Flag Football
- ☐ American Tackle Football
- ☐ Baseball
- ☐ Basketball
- ☐ Cheerleading
- ☐ Cross Country
- ☐ Field Hockey
- ☐ Golf
- ☐ Gymnastics
- ☐ Ice Hockey
- ☐ Lacrosse
- ☐ Rugby
- ☐ Soccer
- ☐ Softball
- ☐ Swimming
- ☐ Tennis
- ☐ Track & Field
- ☐ Volleyball
- ☐ Water Polo
- ☐ Wrestling
- ☐ ________ Other Sport 1
- ☐ ________ Other Sport 2
- ☐ ________ Other Sport 3

Did you have any physical limitations that prevented you from playing on an organized sports team in High School (Grade 9 through Grade 12)?

- Yes
- No
- I prefer not to answer

Note. The following questions are in regards to your participation in sports in High School (Grade 9 through Grade 12).

In which grades did you play baseball? Please select all that apply. You may provide your best estimate if you are not exactly sure.

- Grade 9
- Grade 10
- Grade 11
- Grade 12

Please select all that apply in regards to your participation in baseball. You may provide your best estimate if you are not exactly sure.

- My team traveled for games or tournaments.
- My team participated in playoffs.
- My team won a championship.
- None of the above

On average, how many hours per week did you spend practicing baseball with the team? You may provide your best estimate if you are not exactly

sure.

- 1-4 hours
- 5-9 hours
- 10-14 hours
- 15-19 hours
- 20+ hours

On average, how many hours per week did you spend playing in baseball games? You may provide your best estimate if you are not exactly sure.

- 1-4 hours
- 5-9 hours
- 10-14 hours
- 15-19 hours
- 20+ hours

Please select all positions you played in. You may provide your best estimate if you are not exactly sure.

- Pitcher
- Catcher
- Baseman/Shortstop
- Outfielder
- Other
- None of the above

Please select all that apply in regards to the types of leagues you played baseball in. You may provide your best estimate if you are not exactly sure.

- ☐ I participated in a competitive, school based league
- ☐ I participated in a competitive, non-school based league
- ☐ I participated in a club, recreational, or intramural league at school
- ☐ I participated in a club, recreational, or intramural league outside of school
- ☐ [ ] Other
- ☐ None of the above

In which grades did you play basketball? Please select all that apply. You may provide your best estimate if you are not exactly sure.

- ☐ Grade 9
- ☐ Grade 10
- ☐ Grade 11
- ☐ Grade 12

Please select all that apply in regards to your participation in basketball. You may provide your best estimate if you are not exactly sure.

- ☐ My team traveled for games or tournaments.
- ☐ My team participated in playoffs.
- ☐ My team won a championship.
- ☐ None of the above

On average, how many hours per week did you spend practicing basketball with the team? You may provide your best estimate if you are not exactly sure.

- ○ 1-4 hours
- ○ 5-9 hours
- ○ 10-14 hours
- ○ 15-19 hours
- ○ 20+ hours

On average, how many hours per week did you spend playing in basketball games? You may provide your best estimate if you are not exactly sure.

- ○ 1-4 hours
- ○ 5-9 hours
- ○ 10-14 hours
- ○ 15-19 hours
- ○ 20+ hours

Please select all positions you played in. You may provide your best estimate if you are not exactly sure.

- ☐ Guard
- ☐ Forward
- ☐ Center
- ☐ [ ] Other
- ☐ None of the above

Please select all that apply in regards to the types of leagues you played basketball in. You may provide your best estimate if you are not exactly sure.

- ☐ I participated in a competitive, school based league
- ☐ I participated in a competitive, non-school based league
- ☐ I participated in a club, recreational, or intramural league at school
- ☐ I participated in a club, recreational, or intramural league outside of school
- ☐ [ ] Other
- ☐ None of the above

In which grades did you participate in cheerleading? Please select all that apply. You may provide your best estimate if you are not exactly sure.

- ☐ Grade 9
- ☐ Grade 10
- ☐ Grade 11
- ☐ Grade 12

Please select all that apply in regards to your participation in cheerleading. You may provide your best estimate if you are not exactly sure.

- ☐ I traveled for cheerleading tournaments.
- ☐ My team won a cheerleading tournament.
- ☐ None of the above

On average, how many hours per week did you spend practicing cheerleading? You may provide your best estimate if you are not exactly sure.

- ○ 1-4 hours
- ○ 5-9 hours
- ○ 10-14 hours
- ○ 15-19 hours
- ○ 20+ hours

Please select all that apply in regards to the types of leagues you participated in cheerleading. You may provide your best estimate if you are not exactly sure.

- ☐ I participated in a competitive, school based league
- ☐ I participated in a competitive, non-school based league
- ☐ I participated in a club, recreational, or intramural league at school
- ☐ I participated in a club, recreational, or intramural league outside of school
- ☐ [ ] Other
- ☐ None of the above

In which grades did you participate in cross country? Please select all that apply. You may provide your best estimate if you are not exactly sure.

- ☐ Grade 9
- ☐ Grade 10
- ☐ Grade 11
- ☐ Grade 12

Please select all that apply in regards to your participation in cross country. You may provide your best estimate if you are not exactly sure.

- ☐ I traveled for meets or tournaments.
- ☐ I or my team won a tournament.
- ☐ None of the above

On average, how many hours per week did you spend practicing cross country with the team? You may provide your best estimate if you are not exactly sure.

- ◯ 1-4 hours
- ◯ 5-9 hours
- ◯ 10-14 hours
- ◯ 15-19 hours
- ◯ 20+ hours

On average, how many hours per week did you spend participating in cross country meets? You may provide your best estimate if you are not exactly sure.

- ◯ 1-4 hours
- ◯ 5-9 hours
- ◯ 10-14 hours
- ◯ 15-19 hours
- ◯ 20+ hours

Please select all that apply in regards to the types of leagues you participated in cross country. You may provide your best estimate if you are not exactly sure.

- ☐ I participated in a competitive, school based league
- ☐ I participated in a competitive, non-school based league
- ☐ I participated in a club, recreational, or intramural league at school
- ☐ I participated in a club, recreational, or intramural league outside of school
- ☐ [ ] Other
- ☐ None of the above

In which grades did you participate in field hockey? Please select all that apply. You may provide your best estimate if you are not exactly sure.

- ☐ Grade 9
- ☐ Grade 10
- ☐ Grade 11
- ☐ Grade 12

Please select all that apply in regards to your participation in field hockey. You may provide your best estimate if you are not exactly sure.

- ☐ My team traveled for games or tournaments.
- ☐ My team participated in playoffs.
- ☐ My team won a championship.
- ☐ None of the above

On average, how many hours per week did you spend practicing field hockey with the team? You may provide your best estimate if you are not exactly sure.

- ◯ 1-4 hours
- ◯ 5-9 hours
- ◯ 10-14 hours
- ◯ 15-19 hours
- ◯ 20+ hours

On average, how many hours per week did you spend playing in field hockey games? You may provide your best estimate if you are not exactly sure.

- ◯ 1-4 hours
- ◯ 5-9 hours
- ◯ 10-14 hours
- ◯ 15-19 hours
- ◯ 20+ hours

Please select all positions you played in. You may provide your best estimate if you are not exactly sure.

- ☐ Left/right Defense
- ☐ Left/right Wing
- ☐ Center
- ☐ Goalie
- ☐ [ ] Other

- ☐ None of the above

Please select all that apply in regards to the types of leagues you played field hockey in. You may provide your best estimate if you are not exactly sure.

- ☐ School LeagueI participated in a competitive, school based league
- ☐ I participated in a competitive, non-school based league
- ☐ I participated in a club, recreational, or intramural league at school
- ☐ I participated in a club, recreational, or intramural league outside of school
- ☐ ________ Other
- ☐ None of the above

In which grades did you participate in American tackle football? Please select all that apply. You may provide your best estimate if you are not exactly sure.

- ☐ Grade 9
- ☐ Grade 10
- ☐ Grade 11
- ☐ Grade 12

Please select all that apply in regards to your participation in American tackle football. You may provide your best estimate if you are not exactly sure.

- ☐ My team traveled for games or tournaments.

- ☐ My team participated in playoffs.
- ☐ My team won a championship.
- ☐ None of the above

On average, how many hours per week did you spend practicing American tackle football with the team? You may provide your best estimate if you are not exactly sure.

- ○ 1-4 hours
- ○ 5-9 hours
- ○ 10-14 hours
- ○ 15-19 hours
- ○ 20+ hours

On average, how many hours per week did you spend playing in American tackle football games? You may provide your best estimate if you are not exactly sure.

- ○ 1-4 hours
- ○ 5-9 hours
- ○ 10-14 hours
- ○ 15-19 hours
- ○ 20+ hours

Please select all positions you played in. You may provide your best estimate if you are not exactly sure.

- ☐ Quarterback

- ☐ Running Back
- ☐ Fullback
- ☐ Offensive Line
- ☐ Wide Receiver
- ☐ Tight End
- ☐ Defensive Line
- ☐ Linebacker
- ☐ Cornerback
- ☐ Safety
- ☐ Kicker/Punter
- ☐ Participation in kick off return
- ☐ Long Snapper
- ☐ ________ Other
- ☐ None of the above

Please select all that apply in regards to the types of leagues you played American tackle football in. You may provide your best estimate if you are not exactly sure.

- ☐ I participated in a competitive, school based league
- ☐ I participated in a competitive, non-school based league
- ☐ I participated in a club, recreational, or intramural league at school
- ☐ I participated in a club, recreational, or intramural league outside of school
- ☐ ________ Other
- ☐ None of the above

In which grades did you participate in American flag football? Please select all that apply. You may provide your best estimate if you are not exactly sure.

- ☐ Grade 9
- ☐ Grade 10
- ☐ Grade 11
- ☐ Grade 12

Please select all that apply in regards to your participation in American flag football. You may provide your best estimate if you are not exactly sure.

- ☐ My team traveled for games or tournaments.
- ☐ My team participated in playoffs.
- ☐ My team won a championship.
- ☐ None of the above

On average, how many hours per week did you spend practicing American flag football with the team? You may provide your best estimate if you are not exactly sure.

- ○ 1-4 hours
- ○ 5-9 hours
- ○ 10-14 hours
- ○ 15-19 hours
- ○ 20+ hours

On average, how many hours per week did you spend playing in American flag football games? You may provide your best estimate if you are not exactly sure.

- ○ 1-4 hours
- ○ 5-9 hours
- ○ 10-14 hours
- ○ 15-19 hours
- ○ 20+ hours

Please select all positions you played in. You may provide your best estimate if you are not exactly sure.

- ☐ Quarterback
- ☐ Running Back
- ☐ Fullback
- ☐ Offensive Line
- ☐ Wide Receiver
- ☐ Tight End
- ☐ Defensive Line
- ☐ Linebacker
- ☐ Cornerback
- ☐ Safety
- ☐ Kicker/Punter
- ☐ Participation in kick off return
- ☐ Long Snapper
- ☐ ________ Other
- ☐ None of the above

Please select all that apply in regards to the types of leagues you played American flag football in. You may provide your best estimate if you are not exactly sure.

- ☐ I participated in a competitive, school based league
- ☐ I participated in a competitive, non-school based league
- ☐ I participated in a club, recreational, or intramural league at school
- ☐ I participated in a club, recreational, or intramural league outside of school
- ☐ [ ] Other
- ☐ None of the above

In which grades did you participate in golf? Please select all that apply. You may provide your best estimate if you are not exactly sure.

- ☐ Grade 9
- ☐ Grade 10
- ☐ Grade 11
- ☐ Grade 12

Please select all that apply in regards to your participation in golf. You may provide your best estimate if you are not exactly sure.

- ☐ My team traveled for matches or tournaments.
- ☐ I or my team won a tournament.
- ☐ None of the above

On average, how many hours per week did you spend practicing golf with the team? You may provide your best estimate if you are not exactly sure.

- ○ 1-4 hours
- ○ 5-9 hours
- ○ 10-14 hours
- ○ 15-19 hours
- ○ 20+ hours

On average, how many hours per week did you spend participating in golf matches? You may provide your best estimate if you are not exactly sure.

- ○ 1-4 hours
- ○ 5-9 hours
- ○ 10-14 hours
- ○ 15-19 hours
- ○ 20+ hours

Please select all that apply in regards to the types of leagues you played golf in. You may provide your best estimate if you are not exactly sure.

- ☐ I participated in a competitive, school based league
- ☐ I participated in a competitive, non-school based league
- ☐ I participated in a club, recreational, or intramural league at school
- ☐ I participated in a club, recreational, or intramural league outside of school
- ☐ [ ] Other
- ☐ None of the above

In which grades did you participate in gymnastics? Please select all that apply. You may provide your best estimate if you are not exactly sure.

- ☐ Grade 9
- ☐ Grade 10
- ☐ Grade 11
- ☐ Grade 12

Please select all that apply in regards to your participation in gymnastics. You may provide your best estimate if you are not exactly sure.

- ☐ My team traveled for meets or tournaments.
- ☐ I or my team won a tournament.
- ☐ None of the above

On average, how many hours per week did you spend practicing gymnastics with the team? You may provide your best estimate if you are not exactly sure.

- ○ 1-4 hours
- ○ 5-9 hours
- ○ 10-14 hours
- ○ 15-19 hours
- ○ 20+ hours

On average, how many hours per week did you spend participating in gymnastics meets?

- 1-4 hours
- 5-9 hours
- 10-14 hours
- 15-19 hours
- 20+ hours

Please select all that apply in regards to the types of leagues you participated in gymnastics. You may provide your best estimate if you are not exactly sure.

- I participated in a competitive, school based league
- I participated in a competitive, non-school based league
- I participated in a club, recreational, or intramural league at school
- I participated in a club, recreational, or intramural league outside of school
- [ ] Other
- None of the above

In which grades did you participate in ice hockey? Please select all that apply. You may provide your best estimate if you are not exactly sure.

- Grade 9
- Grade 10
- Grade 11
- Grade 12

Please select all that apply in regards to your participation in ice hockey. You may provide your best estimate if you are not exactly sure.

- ☐ My team traveled for games or tournaments.
- ☐ My team participated in playoffs.
- ☐ My team won a championship.
- ☐ None of the above

On average, how many hours per week did you spend practicing ice hockey with the team? You may provide your best estimate if you are not exactly sure.

- ◯ 1-4 hours
- ◯ 5-9 hours
- ◯ 10-14 hours
- ◯ 15-19 hours
- ◯ 20+ hours

On average, how many hours per week did you spend playing in ice hockey games? You may provide your best estimate if you are not exactly sure.

- ◯ 1-4 hours
- ◯ 5-9 hours
- ◯ 10-14 hours
- ◯ 15-19 hours
- ◯ 20+ hours

Please select all positions you played in. You may provide your best estimate if you are not exactly sure.

- ☐ Left/right Defense
- ☐ Left/right Wing
- ☐ Center
- ☐ Goalie
- ☐ ________ Other
- ☐ None of the above

Please select all that apply in regards to the types of leagues you played ice hockey in. You may provide your best estimate if you are not exactly sure.

- ☐ I participated in a competitive, school based league
- ☐ I participated in a competitive, non-school based league
- ☐ I participated in a club, recreational, or intramural league at school
- ☐ I participated in a club, recreational, or intramural league outside of school
- ☐ ________ Other
- ☐ None of the above

In which grades did you participate in lacrosse? Please select all that apply. You may provide your best estimate if you are not exactly sure.

- ☐ Grade 9
- ☐ Grade 10
- ☐ Grade 11
- ☐ Grade 12

Please select all that apply in regards to your participation in lacrosse. You may provide your best estimate if you are not exactly sure.

- ☐ My team traveled for games or tournaments.
- ☐ My team participated in playoffs.
- ☐ My team won a championship.
- ☐ None of the above

On average, how many hours per week did you spend practicing lacrosse with the team? You may provide your best estimate if you are not exactly sure.

- ◯ 1-4 hours
- ◯ 5-9 hours
- ◯ 10-14 hours
- ◯ 15-19 hours
- ◯ 20+ hours

On average, how many hours per week did you spend playing in lacrosse games? You may provide your best estimate if you are not exactly sure.

- ◯ 1-4 hours
- ◯ 5-9 hours
- ◯ 10-14 hours
- ◯ 15-19 hours
- ◯ 20+ hours

Please select all positions you played in. You may provide your best estimate if you are not exactly sure.

- [ ] Midfielder
- [ ] Attacker
- [ ] Goalie
- [ ] Defenseman
- [ ] ______ Other
- [ ] None of the above

Please select all that apply in regards to the types of leagues you played lacrosse in. You may provide your best estimate if you are not exactly sure.

- [ ] I participated in a competitive, school based league
- [ ] I participated in a competitive, non-school based league
- [ ] I participated in a club, recreational, or intramural league at school
- [ ] I participated in a club, recreational, or intramural league outside of school
- [ ] ______ Other
- [ ] None of the above

In which grades did you participate in rugby? Please select all that apply. You may provide your best estimate if you are not exactly sure.

- [ ] Grade 9
- [ ] Grade 10
- [ ] Grade 11
- [ ] Grade 12

Please select all that apply in regards to your participation in rugby. You may provide your best estimate if you are not exactly sure.

- ☐ My team traveled for games or tournaments.
- ☐ My team participated in playoffs.
- ☐ My team won a championship.
- ☐ None of the above

On average, how many hours per week did you spend practicing rugby with the team? You may provide your best estimate if you are not exactly sure.

- ○ 1-4 hours
- ○ 5-9 hours
- ○ 10-14 hours
- ○ 15-19 hours
- ○ 20+ hours

On average, how many hours per week did you spend playing in rugby games? You may provide your best estimate if you are not exactly sure.

- ○ 1-4 hours
- ○ 5-9 hours
- ○ 10-14 hours
- ○ 15-19 hours
- ○ 20+ hours

Please select all positions you played in. You may provide your best estimate if you are not exactly sure.

- ☐ Forward
- ☐ Back
- ☐ [ ] Other
- ☐ None of the above

Please select all that apply in regards to the types of leagues you played rugby in. You may provide your best estimate if you are not exactly sure.

- ☐ I participated in a competitive, school based league
- ☐ I participated in a competitive, non-school based league
- ☐ I participated in a club, recreational, or intramural league at school
- ☐ I participated in a club, recreational, or intramural league outside of school
- ☐ [ ] Other
- ☐ None of the above

In which grades did you participate in soccer? Please select all that apply. You may provide your best estimate if you are not exactly sure.

- ☐ Grade 9
- ☐ Grade 10
- ☐ Grade 11
- ☐ Grade 12

Please select all that apply in regards to your participation in soccer. You may provide your best estimate if you are not exactly sure.

- ☐ My team traveled for games or tournaments.
- ☐ My team participated in playoffs.
- ☐ My team won a championship.
- ☐ None of the above

On average, how many hours per week did you spend practicing soccer with the team? You may provide your best estimate if you are not exactly sure.

- ○ 1-4 hours
- ○ 5-9 hours
- ○ 10-14 hours
- ○ 15-19 hours
- ○ 20+ hours

On average, how many hours per week did you spend playing in soccer games? You may provide your best estimate if you are not exactly sure.

- ○ 1-4 hours
- ○ 5-9 hours
- ○ 10-14 hours
- ○ 15-19 hours
- ○ 20+ hours

Please select all positions you played in. You may provide your best estimate if you are not exactly sure.

- ☐ Goalkeeper
- ☐ Fullback
- ☐ Center Back
- ☐ Defending/Holding Midfielder
- ☐ Midfielder/Winger
- ☐ Central/Box-to-Box Midfielder
- ☐ Striker
- ☐ Attacking Midfielder/Playmaker
- ☐ ________ Other
- ☐ None of the above

Please select all that apply in regards to the types of leagues you played soccer in. You may provide your best estimate if you are not exactly sure.

- ☐ I participated in a competitive, school based league
- ☐ I participated in a competitive, non-school based league
- ☐ I participated in a club, recreational, or intramural league at school
- ☐ I participated in a club, recreational, or intramural league outside of school
- ☐ ________ Other
- ☐ None of the above

In which grades did you participate in softball? Please select all that apply. You may provide your best estimate if you are not exactly sure.

- ☐ Grade 9
- ☐ Grade 10
- ☐ Grade 11
- ☐ Grade 12

Please select all that apply in regards to your participation in softball. You may provide your best estimate if you are not exactly sure.

- ☐ My team traveled for games or tournaments.
- ☐ My team participated in playoffs.
- ☐ My team won a championship.
- ☐ None of the above

On average, how many hours per week did you spend practicing softball with the team?

- ◯ 1-4 hours
- ◯ 5-9 hours
- ◯ 10-14 hours
- ◯ 15-19 hours
- ◯ 20+ hours

On average, how many hours per week did you spend playing in softball games? You may provide your best estimate if you are not exactly sure.

- ◯ 1-4 hours
- ◯ 5-9 hours
- ◯ 10-14 hours

- 15-19 hours
- 20+ hours

Please select all positions you played in. You may provide your best estimate if you are not exactly sure.

- Pitcher
- Catcher
- Baseman/Shortstop
- Outfielder
- Other
- None of the above

Please select all that apply in regards to the types of leagues you played softball in. You may provide your best estimate if you are not exactly sure.

- I participated in a competitive, school based league
- I participated in a competitive, non-school based league
- I participated in a club, recreational, or intramural league at school
- I participated in a club, recreational, or intramural league outside of school
- Other
- None of the above

In which grades did you participate in swimming? Please select all that apply. You may provide your best estimate if you are not exactly sure.

- ☐ Grade 9
- ☐ Grade 10
- ☐ Grade 11
- ☐ Grade 12

Please select all that apply in regards to your participation in swimming. You may provide your best estimate if you are not exactly sure.

- ☐ My team traveled for meets or tournaments.
- ☐ I or my team won a tournament.
- ☐ None of the above

On average, how many hours per week did you spend practicing swimming with the team? You may provide your best estimate if you are not exactly sure.

- ○ 1-4 hours
- ○ 5-9 hours
- ○ 10-14 hours
- ○ 15-19 hours
- ○ 20+ hours

On average, how many hours per week did you spend participating in swim meets? You may provide your best estimate if you are not exactly sure.

- ○ 1-4 hours
- ○ 5-9 hours
- ○ 10-14 hours

- ◯ 15-19 hours
- ◯ 20+ hours

Please select all that apply in regards to the types of leagues you participated in swimming. You may provide your best estimate if you are not exactly sure.

- ☐ I participated in a competitive, school based league
- ☐ I participated in a competitive, non-school based league
- ☐ I participated in a club, recreational, or intramural league at school
- ☐ I participated in a club, recreational, or intramural league outside of school
- ☐ [ ] Other
- ☐ None of the above

In which grades did you participate in tennis? Please select all that apply. You may provide your best estimate if you are not exactly sure.

- ☐ Grade 9
- ☐ Grade 10
- ☐ Grade 11
- ☐ Grade 12

Please select all that apply in regards to your participation in tennis. You may provide your best estimate if you are not exactly sure.

- ☐ My team traveled for meets or tournaments.
- ☐ I or my team won a tournament.

☐ None of the above

On average, how many hours per week did you spend practicing tennis with the team? You may provide your best estimate if you are not exactly sure.

○ 1-4 hours
○ 5-9 hours
○ 10-14 hours
○ 15-19 hours
○ 20+ hours

On average, how many hours per week did you spend participating in tennis matches? You may provide your best estimate if you are not exactly sure.

○ 1-4 hours
○ 5-9 hours
○ 10-14 hours
○ 15-19 hours
○ 20+ hours

Please select all that apply in regards to the types of leagues you played tennis in. You may provide your best estimate if you are not exactly sure.

☐ I participated in a competitive, school based league
☐ I participated in a competitive, non-school based league
☐ I participated in a club, recreational, or intramural league at school
☐ I participated in a club, recreational, or intramural league outside of school

- ☐ [ ] Other
- ☐ None of the above

In which grades did you participate in track and field? Please select all that apply. You may provide your best estimate if you are not exactly sure.

- ☐ Grade 9
- ☐ Grade 10
- ☐ Grade 11
- ☐ Grade 12

Please select all that apply in regards to your participation in track and field. You may provide your best estimate if you are not exactly sure.

- ☐ I or my team traveled for meets or tournaments.
- ☐ I or my team won a tournament.
- ☐ None of the above

On average, how many hours per week did you spend practicing track and field with the team? You may provide your best estimate if you are not exactly sure.

- ○ 1-4 hours
- ○ 5-9 hours
- ○ 10-14 hours
- ○ 15-19 hours
- ○ 20+ hours

On average, how many hours per week did you spend participating in track and field meets?

- ◯ 1-4 hours
- ◯ 5-9 hours
- ◯ 10-14 hours
- ◯ 15-19 hours
- ◯ 20+ hours

Please select all that apply in regards to the types of leagues you participated in track and field. You may provide your best estimate if you are not exactly sure.

- ☐ I participated in a competitive, school based league
- ☐ I participated in a competitive, non-school based league
- ☐ I participated in a club, recreational, or intramural league at school
- ☐ I participated in a club, recreational, or intramural league outside of school
- ☐ [ ] Other
- ☐ None of the above

In which grades did you participate in volleyball? Please select all that apply. You may provide your best estimate if you are not exactly sure.

- ☐ Grade 9
- ☐ Grade 10
- ☐ Grade 11
- ☐ Grade 12

Please select all that apply in regards to your participation in volleyball. You may provide your best estimate if you are not exactly sure.

- ☐ My team traveled for games or tournaments.
- ☐ My team participated in playoffs.
- ☐ My team won a championship.
- ☐ None of the above

On average, how many hours per week did you spend practicing volleyball with the team? You may provide your best estimate if you are not exactly sure.

- ◯ 1-4 hours
- ◯ 5-9 hours
- ◯ 10-14 hours
- ◯ 15-19 hours
- ◯ 20+ hours

On average, how many hours per week did you spend playing in volleyball games? You may provide your best estimate if you are not exactly sure.

- ◯ 1-4 hours
- ◯ 5-9 hours
- ◯ 10-14 hours
- ◯ 15-19 hours
- ◯ 20+ hours

Please select all positions you played in. You may provide your best estimate if you are not exactly sure.

- [ ] Left/right Front
- [ ] Middle Front
- [ ] Left/right Back
- [ ] Middle Back
- [ ] ________ Other
- [ ] None of the above

Please select all that apply in regards to the types of leagues you played volleyball in. You may provide your best estimate if you are not exactly sure.

- [ ] I participated in a competitive, school based league
- [ ] I participated in a competitive, non-school based league
- [ ] I participated in a club, recreational, or intramural league at school
- [ ] I participated in a club, recreational, or intramural league outside of school
- [ ] ________ Other
- [ ] None of the above

In which grades did you participate in water polo? Please select all that apply. You may provide your best estimate if you are not exactly sure.

- [ ] Grade 9
- [ ] Grade 10
- [ ] Grade 11

☐ Grade 12

Please select all that apply in regards to your participation in water polo. You may provide your best estimate if you are not exactly sure.

☐ My team traveled for games or tournaments.
☐ My team participated in playoffs.
☐ My team won a championship.
☐ None of the above

On average, how many hours per week did you spend practicing water polo with the team? You may provide your best estimate if you are not exactly sure.

○ 1-4 hours
○ 5-9 hours
○ 10-14 hours
○ 15-19 hours
○ 20+ hours

On average, how many hours per week did you spend playing in water polo matches? You may provide your best estimate if you are not exactly sure.

○ 1-4 hours
○ 5-9 hours
○ 10-14 hours
○ 15-19 hours

- 20+ hours

Please select all positions you played in. You may provide your best estimate if you are not exactly sure.

- Goalkeeper
- Defensive Specialist
- Driver
- Two-Meter Specialist
- Other
- None of the above

Please select all that apply in regards to the types of leagues you played water polo in. You may provide your best estimate if you are not exactly sure.

- I participated in a competitive, school based league
- I participated in a competitive, non-school based league
- I participated in a club, recreational, or intramural league at school
- I participated in a club, recreational, or intramural league outside of school
- Other
- None of the above

In which grades did you participate in wrestling? Please select all that apply. You may provide your best estimate if you are not exactly sure.

- ☐ Grade 9
- ☐ Grade 10
- ☐ Grade 11
- ☐ Grade 12

Please select all that apply in regards to your participation in wrestling. You may provide your best estimate if you are not exactly sure.

- ☐ My team traveled for games or tournaments.
- ☐ I or my team won a tournament.
- ☐ None of the above

On average, how many hours per week did you spend practicing wrestling with the team? You may provide your best estimate if you are not exactly sure.

- ○ 1-4 hours
- ○ 5-9 hours
- ○ 10-14 hours
- ○ 15-19 hours
- ○ 20+ hours

On average, how many hours per week did you spend participating in wrestling matches? You may provide your best estimate if you are not exactly sure.

- ○ 1-4 hours
- ○ 5-9 hours

- ◯ 10-14 hours
- ◯ 15-19 hours
- ◯ 20+ hours

Please select all that apply in regards to the types of leagues you participated in wrestling. You may provide your best estimate if you are not exactly sure.

- ☐ I participated in a competitive, school based league
- ☐ I participated in a competitive, non-school based league
- ☐ I participated in a club, recreational, or intramural league at school
- ☐ I participated in a club, recreational, or intramural league outside of school
- ☐ [ ] Other
- ☐ None of the above

In which grades did you participate in ${q://QID218/ChoiceTextEntryValue/21}? Please select all that apply. You may provide your best estimate if you are not exactly sure.

- ☐ Grade 9
- ☐ Grade 10
- ☐ Grade 11
- ☐ Grade 12

Please select all that apply in regards to your participation in ${q://QID218/ChoiceTextEntryValue/21}. You may provide your best estimate if you are not exactly sure.

- ☐ My team traveled for games or tournaments.
- ☐ My team participated in playoffs.
- ☐ My team won a championship.
- ☐ None of the above

On average, how many hours per week did you spend practicing ${q://QID218/ChoiceTextEntryValue/21} with the team? You may provide your best estimate if you are not exactly sure.

- ○ 1-4 hours
- ○ 5-9 hours
- ○ 10-14 hours
- ○ 15-19 hours
- ○ 20+ hours

On average, how many hours per week did you spend playing in ${q://QID218/ChoiceTextEntryValue/21} games? You may provide your best estimate if you are not exactly sure.

- ○ 1-4 hours
- ○ 5-9 hours
- ○ 10-14 hours
- ○ 15-19 hours
- ○ 20+ hours

Please indicate the primary position you played while playing ${q://QID218/ChoiceTextEntryValue/21}. You may provide your best

estimate if you are not exactly sure.

Please select all that apply in regards to the types of leagues you played ${q://QID218/ChoiceTextEntryValue/21} in. You may provide your best estimate if you are not exactly sure.

- ☐ I participated in a competitive, school based league
- ☐ I participated in a competitive, non-school based league
- ☐ I participated in a club, recreational, or intramural league at school
- ☐ I participated in a club, recreational, or intramural league outside of school
- ☐ Other
- ☐ Non of the above

In which grades did you participate in ${q://QID218/ChoiceTextEntryValue/22}? Please select all that apply. You may provide your best estimate if you are not exactly sure.

- ☐ Grade 9
- ☐ Grade 10
- ☐ Grade 11
- ☐ Grade 12

Please select all that apply in regards to your participation in ${q://QID218/ChoiceTextEntryValue/22}. You may provide your best estimate if you are not exactly sure.

- [ ] My team traveled for games or tournaments.
- [ ] My team participated in playoffs.
- [ ] My team won a championship.
- [ ] None of the above

On average, how many hours per week did you spend practicing ${q://QID218/ChoiceTextEntryValue/22} with the team? You may provide your best estimate if you are not exactly sure.

- ○ 1-4 hours
- ○ 5-9 hours
- ○ 10-14 hours
- ○ 15-19 hours
- ○ 20+ hours

On average, how many hours per week did you spend playing in ${q://QID218/ChoiceTextEntryValue/22} games? You may provide your best estimate if you are not exactly sure.

- ○ 1-4 hours
- ○ 5-9 hours
- ○ 10-14 hours
- ○ 15-19 hours
- ○ 20+ hours

Please indicate the primary position you played while playing ${q://QID218/ChoiceTextEntryValue/22}. You may provide your best

estimate if you are not exactly sure.

[ ]

Please select all that apply in regards to the types of leagues you played ${q://QID218/ChoiceTextEntryValue/22} in. You may provide your best estimate if you are not exactly sure.

- ☐ I participated in a competitive, school based league
- ☐ I participated in a competitive, non-school based league
- ☐ I participated in a club, recreational, or intramural league at school
- ☐ I participated in a club, recreational, or intramural league outside of school
- ☐ [ ] Other
- ☐ None of the above

In which grades did you participate in ${q://QID218/ChoiceTextEntryValue/23}? Please select all that apply. You may provide your best estimate if you are not exactly sure.

- ☐ Grade 9
- ☐ Grade 10
- ☐ Grade 11
- ☐ Grade 12

Please select all that apply in regards to your participation in ${q://QID218/ChoiceTextEntryValue/23}. You may provide your best estimate if you are not exactly sure.

- [ ] My team traveled for games or tournaments.
- [ ] My team participated in playoffs.
- [ ] My team won a championship.
- [ ] None of the above

On average, how many hours per week did you spend practicing ${q://QID218/ChoiceTextEntryValue/23} with the team? You may provide your best estimate if you are not exactly sure.

- ○ 1-4 hours
- ○ 5-9 hours
- ○ 10-14 hours
- ○ 15-19 hours
- ○ 20+ hours

On average, how many hours per week did you spend playing in ${q://QID218/ChoiceTextEntryValue/23} games? You may provide your best estimate if you are not exactly sure.

- ○ 1-4 hours
- ○ 5-9 hours
- ○ 10-14 hours
- ○ 15-19 hours
- ○ 20+ hours

Please indicate the primary position you played while playing ${q://QID218/ChoiceTextEntryValue/23}. You may provide your best

estimate if you are not exactly sure.

Please select all that apply in regards to the types of leagues you played ${q://QID218/ChoiceTextEntryValue/23} in. You may provide your best estimate if you are not exactly sure.

- ☐ I participated in a competitive, school based league
- ☐ I participated in a competitive, non-school based league
- ☐ I participated in a club, recreational, or intramural league at school
- ☐ I participated in a club, recreational, or intramural league outside of school
- ☐ Other
- ☐ None of the above

Please detail your participation in any other regular athletic participation outside of an organized team in High School (Grade 9 through Grade 12).

College

Please select all sports that you participated in on an organized team at any time during College.

If a sport you participated in is not listed, you may select "Other Sport" and type in up to three other sports.

If you did not participate in any sports, please select "None."

- ☐ None
- ☐ American Flag Football
- ☐ American Tackle Football
- ☐ Baseball
- ☐ Basketball
- ☐ Cheerleading
- ☐ Cross Country
- ☐ Field Hockey
- ☐ Golf
- ☐ Gymnastics
- ☐ Ice Hockey
- ☐ Lacrosse
- ☐ Rugby
- ☐ Soccer
- ☐ Softball
- ☐ Swimming
- ☐ Tennis
- ☐ Track & Field
- ☐ Volleyball
- ☐ Water Polo
- ☐ Wrestling
- ☐ [ ] Other Sport 1

☐ [ ] Other Sport 2

☐ [ ] Other Sport 3

Did you have any physical limitations that prevented you from playing on an organized sports team in College?

- ○ Yes
- ○ No
- ○ I prefer not to answer

Note. The following questions are in regards to your participation in sports in College.

In which grades did you play baseball? Please select all that apply. You may provide your best estimate if you are not exactly sure.

- ☐ Freshman
- ☐ Sophmore
- ☐ Junior
- ☐ Senior

Please select all that apply in regards to your participation in baseball. You may provide your best estimate if you are not exactly sure.

- [ ] My team traveled for games or tournaments.
- [ ] My team participated in playoffs.
- [ ] My team won a championship.
- [ ] None of the above

On average, how many hours per week did you spend practicing baseball with the team? You may provide your best estimate if you are not exactly sure.

- ◯ 1-4 hours
- ◯ 5-9 hours
- ◯ 10-14 hours
- ◯ 15-19 hours
- ◯ 20+ hours

On average, how many hours per week did you spend playing in baseball games? You may provide your best estimate if you are not exactly sure.

- ◯ 1-4 hours
- ◯ 5-9 hours
- ◯ 10-14 hours
- ◯ 15-19 hours
- ◯ 20+ hours

Please select all positions you played in. You may provide your best estimate if you are not exactly sure.

- [ ] Pitcher

- ☐ Catcher
- ☐ Baseman/Shortstop
- ☐ Outfielder
- ☐ [ ] Other
- ☐ None of the above

Please select all that apply in regards to the types of leagues you played baseball in. You may provide your best estimate if you are not exactly sure.

- ☐ I participated in a competitive, school based league
- ☐ I participated in a competitive, non-school based league
- ☐ I participated in a club, recreational, or intramural league at school
- ☐ I participated in a club, recreational, or intramural league outside of school
- ☐ [ ] Other
- ☐ None of the above

In which grades did you play basketball? Please select all that apply. You may provide your best estimate if you are not exactly sure.

- ☐ Freshman
- ☐ Sophmore
- ☐ Junior
- ☐ Senior

Please select all that apply in regards to your participation in basketball. You may provide your best estimate if you are not exactly sure.

- ☐ My team traveled for games or tournaments.
- ☐ My team participated in playoffs.
- ☐ My team won a championship.
- ☐ None of the above

On average, how many hours per week did you spend practicing basketball with the team? You may provide your best estimate if you are not exactly sure.

- ○ 1-4 hours
- ○ 5-9 hours
- ○ 10-14 hours
- ○ 15-19 hours
- ○ 20+ hours

On average, how many hours per week did you spend playing in basketball games? You may provide your best estimate if you are not exactly sure.

- ○ 1-4 hours
- ○ 5-9 hours
- ○ 10-14 hours
- ○ 15-19 hours
- ○ 20+ hours

Please select all positions you played in. You may provide your best estimate if you are not exactly sure.

- ☐ Guard

- ☐ Forward
- ☐ Center
- ☐ [ ] Other
- ☐ None of the above

Please select all that apply in regards to the types of leagues you played basketball in. You may provide your best estimate if you are not exactly sure.

- ☐ I participated in a competitive, school based league
- ☐ I participated in a competitive, non-school based league
- ☐ I participated in a club, recreational, or intramural league at school
- ☐ I participated in a club, recreational, or intramural league outside of school
- ☐ [ ] Other
- ☐ None of the above

In which grades did you participate in cheerleading? Please select all that apply. You may provide your best estimate if you are not exactly sure.

- ☐ Freshman
- ☐ Sophmore
- ☐ Junior
- ☐ Senior

Please select all that apply in regards to your participation in cheerleading. You may provide your best estimate if you are not exactly

sure.

- ☐ I traveled for cheerleading tournaments.
- ☐ My team won a cheerleading tournament.

On average, how many hours per week did you spend practicing cheerleading?

- ○ 1-4 hours
- ○ 5-9 hours
- ○ 10-14 hours
- ○ 15-19 hours
- ○ 20+ hours

Please select all that apply in regards to the types of leagues you participated in cheerleading. You may provide your best estimate if you are not exactly sure.

- ☐ I participated in a competitive, school based league
- ☐ I participated in a competitive, non-school based league
- ☐ I participated in a club, recreational, or intramural league at school
- ☐ I participated in a club, recreational, or intramural league outside of school
- ☐ [ ] Other
- ☐ None of the above

In which grades did you participate in cross country? Please select all that apply. You may provide your best estimate if you are not exactly sure.

- [ ] Freshman
- [ ] Sophmore
- [ ] Junior
- [ ] Senior

Please select all that apply in regards to your participation in cross country. You may provide your best estimate if you are not exactly sure.

- [ ] I traveled for meets or tournaments.
- [ ] I or my team won a tournament.
- [ ] None of the above

On average, how many hours per week did you spend practicing cross country with the team? You may provide your best estimate if you are not exactly sure.

- ○ 1-4 hours
- ○ 5-9 hours
- ○ 10-14 hours
- ○ 15-19 hours
- ○ 20+ hours

On average, how many hours per week did you spend participating in cross country meets? You may provide your best estimate if you are not exactly sure.

- ○ 1-4 hours
- ○ 5-9 hours

- 10-14 hours
- 15-19 hours
- 20+ hours

Please select all that apply in regards to the types of leagues you participated in cross country. You may provide your best estimate if you are not exactly sure.

- I participated in a competitive, school based league
- I participated in a competitive, non-school based league
- I participated in a club, recreational, or intramural league at school
- I participated in a club, recreational, or intramural league outside of school
- Other
- None of the above

In which grades did you participate in field hockey? Please select all that apply. You may provide your best estimate if you are not exactly sure.

- Freshman
- Sophmore
- Junior
- Senior

Please select all that apply in regards to your participation in field hockey. You may provide your best estimate if you are not exactly sure.

- My team traveled for games or tournaments.

- ☐ My team participated in playoffs.
- ☐ My team won a championship.
- ☐ None of the above

On average, how many hours per week did you spend practicing field hockey with the team? You may provide your best estimate if you are not exactly sure.

- ○ 1-4 hours
- ○ 5-9 hours
- ○ 10-14 hours
- ○ 15-19 hours
- ○ 20+ hours

On average, how many hours per week did you spend playing in field hockey games? You may provide your best estimate if you are not exactly sure.

- ○ 1-4 hours
- ○ 5-9 hours
- ○ 10-14 hours
- ○ 15-19 hours
- ○ 20+ hours

Please select all positions you played in. You may provide your best estimate if you are not exactly sure.

- ☐ Left/right Defense

- ☐ Left/right Wing
- ☐ Center
- ☐ Goalie
- ☐ [ ] Other
- ☐ None of the above

Please select all that apply in regards to the types of leagues you played field hockey in. You may provide your best estimate if you are not exactly sure.

- ☐ I participated in a competitive, school based league
- ☐ I participated in a competitive, non-school based league
- ☐ I participated in a club, recreational, or intramural league at school
- ☐ I participated in a club, recreational, or intramural league outside of school
- ☐ [ ] Other
- ☐ None of the above

In which grades did you participate in American tackle football? Please select all that apply. You may provide your best estimate if you are not exactly sure.

- ☐ Freshman
- ☐ Sophmore
- ☐ Junior
- ☐ Senior

Please select all that apply in regards to your participation in American tackle football. You may provide your best estimate if you are not exactly sure.

- ☐ My team traveled for games or tournaments.
- ☐ My team participated in playoffs.
- ☐ My team won a championship.
- ☐ None of the above

On average, how many hours per week did you spend practicing American tackle football with the team? You may provide your best estimate if you are not exactly sure.

- ○ 1-4 hours
- ○ 5-9 hours
- ○ 10-14 hours
- ○ 15-19 hours
- ○ 20+ hours

On average, how many hours per week did you spend playing in American tackle football games? You may provide your best estimate if you are not exactly sure.

- ○ 1-4 hours
- ○ 5-9 hours
- ○ 10-14 hours
- ○ 15-19 hours
- ○ 20+ hours

Please select all positions you played in. You may provide your best estimate if you are not exactly sure.

- ☐ Quarterback
- ☐ Running Back
- ☐ Fullback
- ☐ Offensive Line
- ☐ Wide Receiver
- ☐ Tight End
- ☐ Defensive Line
- ☐ Linebacker
- ☐ Cornerback
- ☐ Safety
- ☐ Kicker/Punter
- ☐ Participation in kick off return
- ☐ Long Snapper
- ☐ [ ] Other
- ☐ None of the above

Please select all that apply in regards to the types of leagues you played American tackle football in. You may provide your best estimate if you are not exactly sure.

- ☐ I participated in a competitive, school based league
- ☐ I participated in a competitive, non-school based league
- ☐ I participated in a club, recreational, or intramural league at school
- ☐ I participated in a club, recreational, or intramural league outside of school
- ☐ [ ] Other

☐ None of the above

In which grades did you participate in American flag football? Please select all that apply. You may provide your best estimate if you are not exactly sure.

☐ Freshman
☐ Sophmore
☐ Junior
☐ Senior

Please select all that apply in regards to your participation in American flag football. You may provide your best estimate if you are not exactly sure.

☐ My team traveled for games or tournaments.
☐ My team participated in playoffs.
☐ My team won a championship.
☐ None of the above

On average, how many hours per week did you spend practicing American flag football with the team? You may provide your best estimate if you are not exactly sure.

○ 1-4 hours
○ 5-9 hours
○ 10-14 hours
○ 15-19 hours
○ 20+ hours

On average, how many hours per week did you spend playing in American flag football games? You may provide your best estimate if you are not exactly sure.

- ◯ 1-4 hours
- ◯ 5-9 hours
- ◯ 10-14 hours
- ◯ 15-19 hours
- ◯ 20+ hours

Please select all positions you played in. You may provide your best estimate if you are not exactly sure.

- ☐ Quarterback
- ☐ Running Back
- ☐ Fullback
- ☐ Offensive Line
- ☐ Wide Receiver
- ☐ Tight End
- ☐ Defensive Line
- ☐ Linebacker
- ☐ Cornerback
- ☐ Safety
- ☐ Kicker/Punter
- ☐ Participation in kick off return
- ☐ Long Snapper
- ☐ ________ Other
- ☐ None of the above

Please select all that apply in regards to the types of leagues you played American flag football in. You may provide your best estimate if you are not exactly sure.

- ☐ I participated in a competitive, school based league
- ☐ I participated in a competitive, non-school based league
- ☐ I participated in a club, recreational, or intramural league at school
- ☐ I participated in a club, recreational, or intramural league outside of school
- ☐ ________ Other
- ☐ None of the above

In which grades did you participate in golf? Please select all that apply. You may provide your best estimate if you are not exactly sure.

- ☐ Freshman
- ☐ Sophmore
- ☐ Junior
- ☐ Senior

Please select all that apply in regards to your participation in golf. You may provide your best estimate if you are not exactly sure.

- ☐ My team traveled for matches or tournaments.
- ☐ I or my team won a tournament.
- ☐ None of the above

On average, how many hours per week did you spend practicing golf with the team? You may provide your best estimate if you are not exactly sure.

- ◯ 1-4 hours
- ◯ 5-9 hours
- ◯ 10-14 hours
- ◯ 15-19 hours
- ◯ 20+ hours

On average, how many hours per week did you spend participating in golf matches? You may provide your best estimate if you are not exactly sure.

- ◯ 1-4 hours
- ◯ 5-9 hours
- ◯ 10-14 hours
- ◯ 15-19 hours
- ◯ 20+ hours

Please select all that apply in regards to the types of leagues you played golf in. You may provide your best estimate if you are not exactly sure.

- ☐ I participated in a competitive, school based league
- ☐ I participated in a competitive, non-school based league
- ☐ I participated in a club, recreational, or intramural league at school
- ☐ I participated in a club, recreational, or intramural league outside of school
- ☐ ______ Other
- ☐ None of the above

In which grades did you participate in gymnastics? Please select all that apply. You may provide your best estimate if you are not exactly sure.

- ☐ Freshman
- ☐ Sophmore
- ☐ Junior
- ☐ Senior

Please select all that apply in regards to your participation in gymnastics. You may provide your best estimate if you are not exactly sure.

- ☐ My team traveled for meets or tournaments.
- ☐ I or my team won a tournament.
- ☐ None of the above

On average, how many hours per week did you spend practicing gymnastics with the team? You may provide your best estimate if you are not exactly sure.

- ○ 1-4 hours
- ○ 5-9 hours
- ○ 10-14 hours
- ○ 15-19 hours
- ○ 20+ hours

On average, how many hours per week did you spend participating in gymnastics meets? You may provide your best estimate if you are not

exactly sure.

- 1-4 hours
- 5-9 hours
- 10-14 hours
- 15-19 hours
- 20+ hours

Please select all that apply in regards to the types of leagues you participated in gymnastics. You may provide your best estimate if you are not exactly sure.

- I participated in a competitive, school based league
- I participated in a competitive, non-school based league
- I participated in a club, recreational, or intramural league at school
- I participated in a club, recreational, or intramural league outside of school
- ______ Other
- None of the above

In which grades did you participate in ice hockey? Please select all that apply. You may provide your best estimate if you are not exactly sure.

- Freshman
- Sophmore
- Junior
- Senior

Please select all that apply in regards to your participation in ice hockey. You may provide your best estimate if you are not exactly sure.

- ☐ My team traveled for games or tournaments.
- ☐ My team participated in playoffs.
- ☐ My team won a championship.
- ☐ None of the above

On average, how many hours per week did you spend practicing ice hockey with the team? You may provide your best estimate if you are not exactly sure.

- ○ 1-4 hours
- ○ 5-9 hours
- ○ 10-14 hours
- ○ 15-19 hours
- ○ 20+ hours

On average, how many hours per week did you spend playing in ice hockey games? You may provide your best estimate if you are not exactly sure.

- ○ 1-4 hours
- ○ 5-9 hours
- ○ 10-14 hours
- ○ 15-19 hours
- ○ 20+ hours

Please select all positions you played in. You may provide your best estimate if you are not exactly sure.

- ☐ Left/right Defense
- ☐ Left/right Wing
- ☐ Center
- ☐ Goalie
- ☐ [ ] Other
- ☐ None of the above

Please select all that apply in regards to the types of leagues you played ice hockey in. You may provide your best estimate if you are not exactly sure.

- ☐ I participated in a competitive, school based league
- ☐ I participated in a competitive, non-school based league
- ☐ I participated in a club, recreational, or intramural league at school
- ☐ I participated in a club, recreational, or intramural league outside of school
- ☐ [ ] Other
- ☐ None of the above

In which grades did you participate in lacrosse? Please select all that apply. You may provide your best estimate if you are not exactly sure.

- ☐ Freshman
- ☐ Sophmore
- ☐ Junior
- ☐ Senior

Please select all that apply in regards to your participation in lacrosse. You may provide your best estimate if you are not exactly sure.

- ☐ My team traveled for games or tournaments.
- ☐ My team participated in playoffs.
- ☐ My team won a championship.
- ☐ None of the above

On average, how many hours per week did you spend practicing lacrosse with the team? You may provide your best estimate if you are not exactly sure.

- ○ 1-4 hours
- ○ 5-9 hours
- ○ 10-14 hours
- ○ 15-19 hours
- ○ 20+ hours

On average, how many hours per week did you spend playing in lacrosse games? You may provide your best estimate if you are not exactly sure.

- ○ 1-4 hours
- ○ 5-9 hours
- ○ 10-14 hours
- ○ 15-19 hours
- ○ 20+ hours

Please select all positions you played in. You may provide your best estimate if you are not exactly sure.

- ☐ Midfielder
- ☐ Attacker
- ☐ Goalie
- ☐ Defenseman
- ☐ [ ] Other
- ☐ None of the above

Please select all that apply in regards to the types of leagues you played lacrosse in. You may provide your best estimate if you are not exactly sure.

- ☐ I participated in a competitive, school based league
- ☐ I participated in a competitive, non-school based league
- ☐ I participated in a club, recreational, or intramural league at school
- ☐ I participated in a club, recreational, or intramural league outside of school
- ☐ [ ] Other
- ☐ None of the above

In which grades did you participate in rugby? Please select all that apply. You may provide your best estimate if you are not exactly sure.

- ☐ Freshman
- ☐ Sophmore
- ☐ Junior
- ☐ Senior

Please select all that apply in regards to your participation in rugby. You may provide your best estimate if you are not exactly sure.

- ☐ My team traveled for games or tournaments.
- ☐ My team participated in playoffs.
- ☐ My team won a championship.
- ☐ None of the above

On average, how many hours per week did you spend practicing rugby with the team? You may provide your best estimate if you are not exactly sure.

- ○ 1-4 hours
- ○ 5-9 hours
- ○ 10-14 hours
- ○ 15-19 hours
- ○ 20+ hours

On average, how many hours per week did you spend playing in rugby games? You may provide your best estimate if you are not exactly sure.

- ○ 1-4 hours
- ○ 5-9 hours
- ○ 10-14 hours
- ○ 15-19 hours
- ○ 20+ hours

Please select all positions you played in. You may provide your best estimate if you are not exactly sure.

- ☐ Forward
- ☐ Back
- ☐ [ ] Other
- ☐ None of the above

Please select all that apply in regards to the types of leagues you played rugby in. You may provide your best estimate if you are not exactly sure.

- ☐ I participated in a competitive, school based league
- ☐ I participated in a competitive, non-school based league
- ☐ I participated in a club, recreational, or intramural league at school
- ☐ I participated in a club, recreational, or intramural league outside of school
- ☐ [ ] Other
- ☐ None of the above

In which grades did you participate in soccer? Please select all that apply. You may provide your best estimate if you are not exactly sure.

- ☐ Freshman
- ☐ Sophmore
- ☐ Junior
- ☐ Senior

Please select all that apply in regards to your participation in soccer. You may provide your best estimate if you are not exactly sure.

- ☐ My team traveled for games or tournaments.
- ☐ My team participated in playoffs.
- ☐ My team won a championship.
- ☐ None of the above

On average, how many hours per week did you spend practicing soccer with the team? You may provide your best estimate if you are not exactly sure.

- ○ 1-4 hours
- ○ 5-9 hours
- ○ 10-14 hours
- ○ 15-19 hours
- ○ 20+ hours

On average, how many hours per week did you spend playing in soccer games? You may provide your best estimate if you are not exactly sure.

- ○ 1-4 hours
- ○ 5-9 hours
- ○ 10-14 hours
- ○ 15-19 hours
- ○ 20+ hours

Please select all positions you played in. You may provide your best estimate if you are not exactly sure.

- ☐ Goalkeeper
- ☐ Fullback
- ☐ Center Back
- ☐ Defending/Holding Midfielder
- ☐ Midfielder/Winger
- ☐ Central/Box-to-Box Midfielder
- ☐ Striker
- ☐ Attacking Midfielder/Playmaker
- ☐ Other
- ☐ None of the above

Please select all that apply in regards to the types of leagues you played soccer in. You may provide your best estimate if you are not exactly sure.

- ☐ I participated in a competitive, school based league
- ☐ I participated in a competitive, non-school based league
- ☐ I participated in a club, recreational, or intramural league at school
- ☐ I participated in a club, recreational, or intramural league outside of school
- ☐ Other
- ☐ None of the above

In which grades did you participate in softball? Please select all that apply. You may provide your best estimate if you are not exactly sure.

- ☐ Freshman
- ☐ Sophmore
- ☐ Junior
- ☐ Senior

Please select all that apply in regards to your participation in softball. You may provide your best estimate if you are not exactly sure.

- ☐ My team traveled for games or tournaments.
- ☐ My team participated in playoffs.
- ☐ My team won a championship.
- ☐ None of the above

On average, how many hours per week did you spend practicing softball with the team? You may provide your best estimate if you are not exactly sure.

- ◯ 1-4 hours
- ◯ 5-9 hours
- ◯ 10-14 hours
- ◯ 15-19 hours
- ◯ 20+ hours

On average, how many hours per week did you spend playing in softball games? You may provide your best estimate if you are not exactly sure.

- ◯ 1-4 hours
- ◯ 5-9 hours

- ○ 10-14 hours
- ○ 15-19 hours
- ○ 20+ hours

Please select all positions you played in. You may provide your best estimate if you are not exactly sure.

- ☐ Pitcher
- ☐ Catcher
- ☐ Baseman/Shortstop
- ☐ Outfielder
- ☐ [ ] Other
- ☐ None of the above

Please select all that apply in regards to the types of leagues you played softball in. You may provide your best estimate if you are not exactly sure.

- ☐ I participated in a competitive, school based league
- ☐ I participated in a competitive, non-school based league
- ☐ I participated in a club, recreational, or intramural league at school
- ☐ I participated in a club, recreational, or intramural league outside of school
- ☐ [ ] Other
- ☐ None of the above

In which grades did you participate in swimming? Please select all that apply. You may provide your best estimate if you are not exactly sure.

- ☐ Freshman
- ☐ Sophmore
- ☐ Junior
- ☐ Senior

Please select all that apply in regards to your participation in swimming. You may provide your best estimate if you are not exactly sure.

- ☐ My team traveled for meets or tournaments.
- ☐ I or my team won a tournament.
- ☐ None of the above

On average, how many hours per week did you spend practicing swimming with the team? You may provide your best estimate if you are not exactly sure.

- ◯ 1-4 hours
- ◯ 5-9 hours
- ◯ 10-14 hours
- ◯ 15-19 hours
- ◯ 20+ hours

On average, how many hours per week did you spend participating in swim meets? You may provide your best estimate if you are not exactly sure.

- ◯ 1-4 hours
- ◯ 5-9 hours
- ◯ 10-14 hours

- 15-19 hours
- 20+ hours

Please select all that apply in regards to the types of leagues you participated in swimming. You may provide your best estimate if you are not exactly sure.

- I participated in a competitive, school based league
- I participated in a competitive, non-school based league
- I participated in a club, recreational, or intramural league at school
- I participated in a club, recreational, or intramural league outside of school
- Other
- None of the above

In which grades did you participate in tennis? Please select all that apply. You may provide your best estimate if you are not exactly sure.

- Freshman
- Sophmore
- Junior
- Senior

Please select all that apply in regards to your participation in tennis. You may provide your best estimate if you are not exactly sure.

- My team traveled for meets or tournaments.
- I or my team won a tournament.

- ☐ None of the above

On average, how many hours per week did you spend practicing tennis with the team? You may provide your best estimate if you are not exactly sure.

- ○ 1-4 hours
- ○ 5-9 hours
- ○ 10-14 hours
- ○ 15-19 hours
- ○ 20+ hours

On average, how many hours per week did you spend participating in tennis matches? You may provide your best estimate if you are not exactly sure.

- ○ 1-4 hours
- ○ 5-9 hours
- ○ 10-14 hours
- ○ 15-19 hours
- ○ 20+ hours

Please select all that apply in regards to the types of leagues you played tennis in. You may provide your best estimate if you are not exactly sure.

- ☐ I participated in a competitive, school based league
- ☐ I participated in a competitive, non-school based league
- ☐ I participated in a club, recreational, or intramural league at school
- ☐ I participated in a club, recreational, or intramural league outside of school

- [ ] [ ] Other
- [ ] None of the above

In which grades did you participate in track and field? Please select all that apply. You may provide your best estimate if you are not exactly sure.

- [ ] Freshman
- [ ] Sophmore
- [ ] Junior
- [ ] Senior

Please select all that apply in regards to your participation in track and field. You may provide your best estimate if you are not exactly sure.

- [ ] I or my team traveled for meets or tournaments.
- [ ] I or my team won a tournament.
- [ ] None of the above

On average, how many hours per week did you spend practicing track and field with the team? You may provide your best estimate if you are not exactly sure.

- ○ 1-4 hours
- ○ 5-9 hours
- ○ 10-14 hours
- ○ 15-19 hours
- ○ 20+ hours

On average, how many hours per week did you spend participating in track and field meets? You may provide your best estimate if you are not exactly sure.

- ◯ 1-4 hours
- ◯ 5-9 hours
- ◯ 10-14 hours
- ◯ 15-19 hours
- ◯ 20+ hours

Please select all that apply in regards to the types of leagues you participated in track and field. You may provide your best estimate if you are not exactly sure.

- ☐ I participated in a competitive, school based league
- ☐ I participated in a competitive, non-school based league
- ☐ I participated in a club, recreational, or intramural league at school
- ☐ I participated in a club, recreational, or intramural league outside of school
- ☐ [ ] Other
- ☐ None of the above

In which grades did you participate in volleyball? Please select all that apply. You may provide your best estimate if you are not exactly sure.

- ☐ Freshman
- ☐ Sophmore
- ☐ Junior

☐ Senior

Please select all that apply in regards to your participation in volleyball. You may provide your best estimate if you are not exactly sure.

☐ My team traveled for games or tournaments.
☐ My team participated in playoffs.
☐ My team won a championship.
☐ None of the above

On average, how many hours per week did you spend practicing volleyball with the team? You may provide your best estimate if you are not exactly sure.

○ 1-4 hours
○ 5-9 hours
○ 10-14 hours
○ 15-19 hours
○ 20+ hours

On average, how many hours per week did you spend playing in volleyball games? You may provide your best estimate if you are not exactly sure.

○ 1-4 hours
○ 5-9 hours
○ 10-14 hours
○ 15-19 hours

- ○ 20+ hours

Please select all positions you played in. You may provide your best estimate if you are not exactly sure.

- ☐ Left/right Front
- ☐ Middle Front
- ☐ Left/right Back
- ☐ Middle Back
- ☐ [ ] Other
- ☐ None of the above

Please select all that apply in regards to the types of leagues you played volleyball in. You may provide your best estimate if you are not exactly sure.

- ☐ I participated in a competitive, school based league
- ☐ I participated in a competitive, non-school based league
- ☐ I participated in a club, recreational, or intramural league at school
- ☐ I participated in a club, recreational, or intramural league outside of school
- ☐ [ ] Other
- ☐ None of the above

In which grades did you participate in water polo? Please select all that apply. You may provide your best estimate if you are not exactly sure.

- ☐ Freshman

- ☐ Sophmore
- ☐ Junior
- ☐ Senior

Please select all that apply in regards to your participation in water polo. You may provide your best estimate if you are not exactly sure.

- ☐ My team traveled for games or tournaments.
- ☐ My team participated in playoffs.
- ☐ My team won a championship.
- ☐ None of the above

On average, how many hours per week did you spend practicing water polo with the team? You may provide your best estimate if you are not exactly sure.

- ◯ 1-4 hours
- ◯ 5-9 hours
- ◯ 10-14 hours
- ◯ 15-19 hours
- ◯ 20+ hours

On average, how many hours per week did you spend playing in water polo matches? You may provide your best estimate if you are not exactly sure.

- ◯ 1-4 hours
- ◯ 5-9 hours

- ○ 10-14 hours
- ○ 15-19 hours
- ○ 20+ hours

Please select all positions you played in. You may provide your best estimate if you are not exactly sure.

- ☐ Goalkeeper
- ☐ Defensive Specialist
- ☐ Driver
- ☐ Two-Meter Specialist
- ☐ [ ] Other
- ☐ None of the above

Please select all that apply in regards to the types of leagues you played water polo in. You may provide your best estimate if you are not exactly sure.

- ☐ I participated in a competitive, school based league
- ☐ I participated in a competitive, non-school based league
- ☐ I participated in a club, recreational, or intramural league at school
- ☐ I participated in a club, recreational, or intramural league outside of school
- ☐ [ ] Other
- ☐ None of the above

In which grades did you participate in wrestling? Please select all that apply. You may provide your best estimate if you are not exactly sure.

- ☐ Freshman
- ☐ Sophmore
- ☐ Junior
- ☐ Senior

Please select all that apply in regards to your participation in wrestling. You may provide your best estimate if you are not exactly sure.

- ☐ My team traveled for games or tournaments.
- ☐ I or my team won a tournament.
- ☐ None of the above

On average, how many hours per week did you spend practicing wrestling with the team? You may provide your best estimate if you are not exactly sure.

- ◯ 1-4 hours
- ◯ 5-9 hours
- ◯ 10-14 hours
- ◯ 15-19 hours
- ◯ 20+ hours

On average, how many hours per week did you spend participating in wrestling matches? You may provide your best estimate if

you are not exactly sure.

- 1-4 hours
- 5-9 hours
- 10-14 hours
- 15-19 hours
- 20+ hours

Please select all that apply in regards to the types of leagues you participated in wrestling. You may provide your best estimate if you are not exactly sure.

- I participated in a competitive, school based league
- I participated in a competitive, non-school based league
- I participated in a club, recreational, or intramural league at school
- I participated in a club, recreational, or intramural league outside of school
- ______ Other
- None of the above

In which grades did you participate in ${q://QID323/ChoiceTextEntryValue/21}? Please select all that apply. You may provide your best estimate if you are not exactly sure.

- Freshman
- Sophmore
- Junior
- Senior

Please select all that apply in regards to your participation in ${q://QID323/ChoiceTextEntryValue/21}. You may provide your best estimate if you are not exactly sure.

- ☐ My team traveled for games or tournaments.
- ☐ My team participated in playoffs.
- ☐ My team won a championship.
- ☐ None of the above

On average, how many hours per week did you spend practicing ${q://QID323/ChoiceTextEntryValue/21} with the team? You may provide your best estimate if you are not exactly sure.

- ◯ 1-4 hours
- ◯ 5-9 hours
- ◯ 10-14 hours
- ◯ 15-19 hours
- ◯ 20+ hours

On average, how many hours per week did you spend playing in ${q://QID323/ChoiceTextEntryValue/21} games? You may provide your best estimate if you are not exactly sure.

- ◯ 1-4 hours
- ◯ 5-9 hours
- ◯ 10-14 hours
- ◯ 15-19 hours
- ◯ 20+ hours

Please indicate the primary position you played while playing ${q://QID323/ChoiceTextEntryValue/21}. You may provide your best estimate if you are not exactly sure.

Please select all that apply in regards to the types of leagues you played ${q://QID323/ChoiceTextEntryValue/21} in. You may provide your best estimate if you are not exactly sure.

- ☐ I participated in a competitive, school based league
- ☐ I participated in a competitive, non-school based league
- ☐ I participated in a club, recreational, or intramural league at school
- ☐ I participated in a club, recreational, or intramural league outside of school
- ☐ Other
- ☐ None of the above

In which grades did you participate in ${q://QID323/ChoiceTextEntryValue/22}? Please select all that apply. You may provide your best estimate if you are not exactly sure.

- ☐ Freshman
- ☐ Sophmore
- ☐ Junior
- ☐ Senior

Please select all that apply in regards to your participation in ${q://QID323/ChoiceTextEntryValue/22}. You may provide your best estimate if you are not exactly sure.

- ☐ My team traveled for games or tournaments.
- ☐ My team participated in playoffs.
- ☐ My team won a championship.
- ☐ None of the above

On average, how many hours per week did you spend practicing ${q://QID323/ChoiceTextEntryValue/22} with the team? You may provide your best estimate if you are not exactly sure.

- ○ 1-4 hours
- ○ 5-9 hours
- ○ 10-14 hours
- ○ 15-19 hours
- ○ 20+ hours

On average, how many hours per week did you spend playing in ${q://QID323/ChoiceTextEntryValue/22} games? You may provide your best estimate if you are not exactly sure.

- ○ 1-4 hours
- ○ 5-9 hours
- ○ 10-14 hours
- ○ 15-19 hours
- ○ 20+ hours

Please indicate the primary position you played while playing ${q://QID323/ChoiceTextEntryValue/22}. You may provide your best estimate if you are not exactly sure.

Please select all that apply in regards to the types of leagues you played ${q://QID323/ChoiceTextEntryValue/22} in. You may provide your best estimate if you are not exactly sure.

- ☐ I participated in a competitive, school based league
- ☐ I participated in a competitive, non-school based league
- ☐ I participated in a club, recreational, or intramural league at school
- ☐ I participated in a club, recreational, or intramural league outside of school
- ☐ Other
- ☐ None of the above

In which grades did you participate in ${q://QID323/ChoiceTextEntryValue/23}? Please select all that apply. You may provide your best estimate if you are not exactly sure.

- ☐ Freshman
- ☐ Sophmore
- ☐ Junior
- ☐ Senior

Please select all that apply in regards to your participation in ${q://QID323/ChoiceTextEntryValue/23}. You may provide your best estimate if you are not exactly sure.

- ☐ My team traveled for games or tournaments.
- ☐ My team participated in playoffs.
- ☐ My team won a championship.
- ☐ None of the above

On average, how many hours per week did you spend practicing ${q://QID323/ChoiceTextEntryValue/23} with the team? You may provide your best estimate if you are not exactly sure.

- ○ 1-4 hours
- ○ 5-9 hours
- ○ 10-14 hours
- ○ 15-19 hours
- ○ 20+ hours

On average, how many hours per week did you spend playing in ${q://QID323/ChoiceTextEntryValue/23} games? You may provide your best estimate if you are not exactly sure.

- ○ 1-4 hours
- ○ 5-9 hours
- ○ 10-14 hours
- ○ 15-19 hours
- ○ 20+ hours

Please indicate the primary position you played while playing ${q://QID323/ChoiceTextEntryValue/23}. You may provide your best estimate if you are not exactly sure.

Please select all that apply in regards to the types of leagues you played ${q://QID323/ChoiceTextEntryValue/23} in. You may provide your best estimate if you are not exactly sure.

- ☐ I participated in a competitive, school based league
- ☐ I participated in a competitive, non-school based league
- ☐ I participated in a club, recreational, or intramural league at school
- ☐ I participated in a club, recreational, or intramural league outside of school
- ☐ Other
- ☐ None of the above

Please detail your participation in any other regular athletic participation outside of an organized team in College.

Powered by Qualtrics
