# Supplementary material for: Retrospective survey of youth sports participation: Development and assessment of reliability using school records
Source: PLoS One. 2021 Sep 17;16(9):e0257487. doi: 10.1371/journal.pone.0257487 (PMC8448309; doi:10.1371/journal.pone.0257487)
Supplement: S1 Text — (RTF) [file pone.0257487.s004.rtf]

Data File 1 Explanation:	Contains raw data on sports participation, including measures of intensity such as years 	played, hours played per week, and team achievements.Data File 2 Explanation:		Contains cleaned data used for analysis.	Self-reported participation confirmed by yearbook/school database: 1	Self-reported participation not confirmed by yearbook/school database: 0	Participation not self-reported but found through yearbook/school database: -1	Participation not self-reported or found through yearbook.school database: NASurvey Explanation:	The question used to assess high school participation in a sport was the following:Q218 Please select all sports that you participated in on an organized team at any time during High School (Grade 9 through Grade 12). You will have the opportunity to describe any additional physical activities you participated in after answering questions about your participation in team sports.   f a sport you participated in is not listed, you may select "Other Sport" and type in up to three other sports.    f you did not participate in any sports, please select "None."▢None  (1) ▢American Flag Football  (24) ▢American Tackle Football  (7) ▢Baseball  (2) ▢Basketball  (3) ▢Cheerleading  (4) ▢Cross Country  (5) ▢Field Hockey  (6) ▢Golf  (8) ▢Gymnastics    (9) ▢Ice Hockey    (10) ▢Lacrosse  (11) ▢Rugby  (12) ▢Soccer  (13) ▢Softball  (14) ▢Swimming  (15) ▢Tennis  (16) ▢Track & Field  (17) ▢Volleyball  (18) ▢Water Polo  (19) ▢Wrestling  (20) ▢Other Sport 1  (21) ________________________________________________▢Other Sport 2  (22) ________________________________________________▢Other Sport 3  (23) ________________________________________________
