# Supplementary material for: Retrospective survey of youth sports participation: Development and assessment of reliability using school records
Source: PLoS One. 2021 Sep 17;16(9):e0257487. doi: 10.1371/journal.pone.0257487 (PMC8448309; doi:10.1371/journal.pone.0257487)
Supplement: Attachment — Submitted filename: Response to Reviewers.docx [file pone.0257487.s005.docx]

**Retrospective survey of youth sports participation: development and validation using school records**

**Manuscript ID: D-20-27226**

**POINT-BY-POINT RESPONSE**

We thank the two anonymous reviewers for their helpful comments on our submitted manuscript. We have addressed each of these comments in our revised manuscript and have provided a point-by-point response below. Note that, in some cases, reviewer comments have been combined, shortened, or separated into comments with a single theme. Reviewer comments are **bold-faced** whereas our responses are in normal font.

**Reviewer 1 Comment 1: This paper attempts to introduce and validate a retrospective survey of sports participation by utilising respondents self-reported sports participation data and validating responses using yearbook and online databases. The introduction to the paper raised some interesting points, specifically around the investigation of physical and psychological risks of long-term sports participation, however no attempt seemed to be made to answer these questions There is also a lack of clarity relating to the methods section, particularly around the items used in the questionnaire – especially what was to be validated and what was not. The data set relating to intensity did not seem to be available. More broadly, it is felt that the aim of the paper and the associated research question is underdeveloped and unclear – the purpose and the need for the survey is questioned. As such, it is believed the paper is not currently suitable for publication. Below is a sectional review of the paper – it is hoped the authors find the more focused comments useful***.*

Thanks for the useful comments. We have edited the paper to clarify that the aim of the paper is to introduce a survey of retrospective sports participation and to validate aspects of the survey. We have edited the abstract and introduction to clarify these aims. For example, we have rewritten the first two lines of the abstract to be**, *“****Many youth participate in sports and it is of interest to understand the impact of youth sports participation on later-life outcomes. However, prospective studies take a long time to complete and retrospective studies may be more practical and time-efficient to address some questions.”* We have edited the introduction and discussion to clarify that the survey is intended to help with investigating physical and psychological risks and benefits of sports participation, but we do not answer questions about the risks and benefits in this paper. We have rewritten the methods section to clarify which responses in the survey were validated and which were not. We have made the data set related to intensity available.

**Reviewer 1 Comment 2: The introduction begins with an opening line, and much of the opening paragraph, highlighting the detrimental effects of repeated head injury, however the authors do not follow this sporting and social dilemma within the survey investigation. The need to undertake work to survey longitudinal data relating to head trauma and sports participation versus the dilemma of the positive effects of sports participation were established on page 1 and 2 – however this did not seem to continue through the remaining sections of the paper. For example, the authors need to clarify why participants were not asked if they suffered concussion during their time at high school. Why was this question not included in the survey – especially when they included questions relating to intensity, which in the end could not be validated?**

We agree with the reviewer that the introduction could be better structured to set up the aims of the study. In response to this comment, we have restructured the introduction to lead with this discussion of youth sports participation, which is the subject of the survey. We did not include survey items on history of brain injury/concussion because validated measures for assessing history of brain injury history are already available. We now include this information in the introduction.

**Reviewer 1 Comment 3: Page 3 paragraph 2, line 1-5: A decline in sports participation was noted, however these figures were not substantiated. For example, if a drop in 2018-19 was recorded what were the previous years figures?**

The number of participants in high school sports declined from 7,980,886 in 2017-2018 to 7,937,491 in 2018-2019. These figures come from the National Federation of State High School Associations. Our revised introduction now includes these figures.

**Reviewer 1 Comment 4. Page 4, 1st paragraph, line 5-7: mixed findings from previous research (and there seems to be numerous studies available) warrants further research investigating the impact of high school sports on concurrent and later-life outcomes. This process of investigation does not seem to be associated with the aims and survey development outlined? It was felt the introduction takes the reader on an important journey and raises important issues, but the path does not lead anywhere.**

**It may be useful for the authors to utilise the introduction in a more focused way – i.e. what the gap in the current literature is and where this paper fits within that. Within this, it would also be useful to offer more detail on how existing survey tools and questionnaires require this improvement.**

We have now restructured the introduction per the reviewers’ suggestion, putting discussion of youth sports participation up front, and also adding detail on the current measures available for assessing prior brain injury. In the penultimate paragraph of the introduction, we directly state the gap in the current literature, and how the survey presented in the paper fills that gap.

**Reviewer 1 Comment 5: What survey items were developed by the authors and what informed their inclusion? How did this compare with other surveys? What are the differences – this will assist in understanding why we need this survey? Sports were included for the potential of concussion/risk to hit on head; however, this was not a question in the survey (see above point). How many items were included in the survey?**

We developed our survey using a series of blocks to assess sports participation at various stages of one's education. This included a block for grades K-6, grades 7-8, grades 9-12, and college. We structured the survey this way to assess the timing and duration of sports participation while inherently collecting information about competitiveness (eg, participation in college sports is likely to be more competitive than grades 7-8 or high school [grades 9-12]). The four blocks (K-6, grades 7-8, grades 9-12, and college) were identical in the sense that the same series of questions and response options were provided. In addition, we included an introductory block to collect demographic information on the respondent (e.g., age, gender, race, ethnicity, educational attainment, and where they attended high school).

The sports included in our survey were derived, in part, from the high school athletics participation survey conducted by the National Federation of State High School Associations. We also took cues from the Life History Survey 2017 of the Health and Retirement Study which assessed whether respondents participated in sports during junior/middle and high school. However, one potential limitation of the aforementioned survey is that it has not been validated. Thus, we developed the survey items for our survey by drawing from these existing surveys in addition to collecting detailed information about high school attendance history which allowed us to validate survey responses using historical yearbook data.

In total, there were 545 survey items but these were based on branching logic; if an individual participated in every listed support at each time point, they would have responded to 545 queries.

**Reviewer 1 Comment 6: In the discussion section it is noted this is indeed a pilot study. It would have been useful to highlight this in the introduction or methods. This can account for the low numbers associated with the paper. Also, as the number of items were not included in the survey development section it is difficult to comment on the sample size. However, it seems, for some sports, a high agreement scores were achieved for low participant numbers. Further clarity is required regarding what participants were agreeing to (the items) in order to achieve full recall after at least 20+ years out of high school. For example, in the ‘weakness’ section of the discussion it states that the intensity data could not be validated – therefore was only a single binary question relating to type of sport and confirmation of participation requested? This is unclear. Did the information contained in High School year books assist in item formation? If it did not, the study assumes all high school yearbooks will contain comparative data. This may be the case if the items used for validation was a simple yes/no to the sports the respondents participated in – but this is not clear.**

We have now highlighted the fact that our study is indeed a pilot study earlier in the abstract and introduction of our paper. We have also added an acknowledgement of the small sample sizes -- see last sentence of first paragraph of the Discussion. The high school yearbooks were not used to design our survey items. Rather, we used the yearbook to validate the survey results.

We have provided further clarity about the survey and what respondents were agreeing to by adding a subsection “Survey” to the Methods Section of our revised manuscript in which we write *“In our survey, subjects were asked to select the sports in which they participated from a list of radio buttons. The survey used a series of blocks to assess sports participation at various stages of one's education. This included a block for grades K-6, grades 7-8, grades 9-12, and college. For each block corresponding to a specific period in the life course, respondents were asked to select the sports in which they participated from a list of radio buttons. Using this, binary variables were constructed for participation in each sport during high school. Respondents also indicated whether, at each specific period, they had any physical limitations that prevented them from participating in sports. For each selected sport at each time period, respondents were asked to report (using radio buttons) the specific grades in which they participated, whether their team (a) traveled for games or tournaments, (b) participated in playoffs, (c) won a championship, or (d) none of the above. Respondents also indicated how many hours per week (0 hours, 1-4 hours, 5-9 hours, 10-14 hours, 15-19 hours, 20+ hours) they spent practicing and playing games for each specific sport, in addition to the position(s) they played.”*

**Reviewer 1 Comment 7: Page 10 line 1: This feels like quite an optimistic statement, mostly due to sample sizes when breaking the findings down to specific sports. In the limitations part (there is no specific limitations section) it is noted that the study provides no way to evaluate the validity of the sports intensity measures – this is a crucial omission, as it does not provide the opportunity to delve deeper into the psychological and physical effects of athletic participation. In addition, the first paragraphs of the study seem to outline what data could or would have been useful to collect, rather than focusing on what was collected. In order to rectify this the authors may wish to consider a restructure the paper. Also, in the limitations (weakness) section the use of a convenience sample was described – this should be noted and acknowledged in the methods section. In the final paragraph there is first mention of ‘pilot study’ – this should have been detailed in the methods section. It may be useful for the authors to restructure the discussion section by having more specific sections. For example, the authors offer some discussion on possible applications, however this could be more focused. In addition, a separate limitations section would also be useful.**

Thanks for these helpful comments. We have qualified the statement originally on Page 10, line 1: “Our study provides preliminary evidence that individuals are reliable reporters of their high school sports-participation, and the survey presented here is one instrument that could be used for this purpose. However, this is a pilot study with small sample sizes and further research is warranted.”

We have edited the first sentence of the “Sample Collection” section of the Methods section to acknowledge that this is a pilot study with a convenience sample: *“A pilot study was conducted to assess the reliability of the survey by constructing a convenience sample that distributed the survey through alumni e-mail lists and social media groups from high schools where authors (D.S. and A.R) are alumni.”*

We have restructured the discussion to have more specific sections and provided a more focused direction of possible applications in our “Future Directions” section.

**Reviewer 2 Comment 1:** **The topic the authors address is a relevant issue in the field and the authors should be commended on their attempt to validate a measure of previous sport participation. The study overall was well written and presented, however there are some concerns that need to be addressed. Specifically the use of Cronbach’s alpha and Cohen’s kappa as appropriate for this study is needed. Previously internal consistency is shown as correlations between different items or sub scales on the same measure not between measures, and inter-rater reliability is between separate observers, RA's etc. using the same measure, again not between measures. Justification for using these methods for a between measures analysis is needed. Any data on hours, years, measures of intensity, as the authors labeled it, is missing from the submission.**

We appreciate the reviewer’s overall assessment of our work. We concede that our use of Cronbach’s alpha and Cohen’s kappa is somewhat nonstandard. In our revised manuscript, we have removed the analysis based on Cohen’s kappa. We have, however, retained the analysis based on Cronbach’s alpha for the following reason. Essentially, we have two ostensible measurements of the single construct, sports participation. Computing Cronbach’s alpha allows us to assess to the extent to which these two measures really are measuring the same thing. We have included this justification in our revised manuscript. We will make a de-identified data file available.

**Reviewer 2 Comment 2: I found it difficult to see the connection of the introduction (particularly the first three paragraphs) to the purpose and results of the paper. While the introduction was clear and well written, the link between the focus on contact sports and concussions as the supporting literature and the validation of the tool itself was missing. I would suggest cutting the first section on contact sports and CTE unless authors can explicitly link it to the validating of the measure on all sport participation. There is literature that highlights a need for a valid measure of previous sport participation, and this would be better suited for the introduction. Further the authors highlight the optimal ways of collecting data (prospective, longitudinal) but then settle on a retrospective method, while they provide some information as to why the other methods aren’t often used, there is no explanation why their retrospective method is used or why it is beneficial.**

We have now restructured the introduction (see R1.2), cutting some of the background on CTE, and adding a more focused discussion of how the present study fills gaps in the literature. We have also added the following to the final paragraph of the introduction,

***“****Retrospective assessment of youth sports participation has the advantage of allowing investigators to capitalize on existing longitudinal datasets that have already tracked cognitive and health outcomes into later-life, when some of the deleterious effects of brain injury are thought to manifest.”*

**Reviewer 2 Comment 3.** **The authors need to explain why photo and yearbook rosters were used over other methods. The authors themselves point out that people could often be away for picture day and year books could have incorrect or missing names. Additionally, depending when the photos were taken athletes could have quit the team soon after and therefore the information would not match. It is an interesting and novel approach, but further justification is needed***.*

We considered several other potential sources against which to validate the self-reported sports participation data. Unfortunately many of them, such as retrospective reports from parents, siblings, coaches, and friends, are subject to the same potential recall biases as the self-reports themselves. In the absence of prospectively collected participation data, yearbooks and school records offer a nearly-contemporaneous source of sports participation that is not subject to recall bias.

That being said, the referee is correct that yearbooks are not 100% reliable. Nevertheless, our preliminary results suggest that there may be considerable agreement between retrospective self-reports and yearbook records.

**Reviewer 2 Comment 4:** **The authors should explain why these atypical approaches are suitable for their purposes, especially on top of the approach of percent agreement. Are these approaches appropriate to use for inter-rater reliability or internal consistency? If so, the authors need to provide examples of when this has been done in the past or why this should be considered an appropriate use of Cronbach’s alpha and Cohen’s kappa.**

In a certain sense, we have two items (yearbook record and self-report) that purport to measure the same thing (sports participation). As a result, we argue that it is useful to consider the internal consistency of these two items and to report the value of Cronbach’s alpha.

**Reviewer 2 Comment 5.** **The authors should provide justification for removing participants who took a long time to complete. Was completing the survey in one time session required? Why was interruption a problem?**

We thank the reviewer for their comments. We wish to make a point of clarification regarding the removal of participants. These respondents were not removed from the analytic sample; we only excluded them to calculate the adjusted mean survey completion time after accounting for outliers. However, we agree that this may be a point of confusion in the manuscript. As such, we removed this comment from the main text.

**Reviewer 2 Comment 6: The purpose of the paper was to validate a measure of sport participation, yet there is no indication of an effort to validate measure of intensity. The authors collected information on hours, year and achievements, but this section was not validated? I suggest the authors explain why they did not validate this section, show that it was validated, or remove it from the paper. The authors did mention that the year-book does not provide measures on intensity but there are other methods for validating this information, or if it does not fit with the year-book method then it should be removed.**

We considered other methods for validating intensity items. Detailed school reports of participation intensity were not available for this purpose. We considered collateral report from either parents or other school sources, however, these reports would have been burdensome to acquire, and we were not at all confident that they would be appropriate measures for validating this information-- that is, the retrospective reports from collaterals would also be subject to memory biases. Hence, we determined that there was no appropriate comparison standard for evaluating the validity of these items in the present study.

Despite this limitation, we have decided to include the intensity data in the manuscript. These data highlight an aspect of the survey that captures more granular information on sports participation, which we believe is a potential strength of the survey. The predictive validity of these data could be established in future work examining relationships between sports participation intensity and outcomes of interest. Such an examination is outside of the scope of the current work, and an important direction for future research.

**Reviewer 2 Comment 7:** **Table 1: Title is misleading, “Interrater Reliability for Survey Responses and School Resources” as this table also shows, internal consistency and percent agreement?**

Thank you for pointing this out. We will rename the table “Percent Agreement and Internal Consistency for Survey Responses and School Resources”

**Reviewer 2 Comment 8:** **Again the authors highlight concerns around using the yearbook information to validate measures “inconsistency between self-reported participation and school records may be due to errors in record keeping, rather than memory failures on the part of the survey respondents” and therefore the authors need to make a stronger argument for use of yearbooks. The discussion highlights the need for information on intensity, but the study fails to validate this information. Such a focus on intensity and consequences of intensity in the discussion seems out of place when measures of intensity were not validated***.*

We appreciate the reviewer’s pointing this out. As we argue in response to Reviewer 2 Comment 3, yearbooks are not subject to the same biases as self-report or collateral interviews with parents, friends, and school officials. The relatively high agreement observed in our pilot study suggests that there may not be substantial errors in record keeping. We have reduced the emphasis on intensity in the discussion of the revised manuscript.

**Reviewer 2 Comment 9: The authors provide the data for the coding and analysis of sports but there is not data with intensity information ?**

We have now included a data file that contains intensity information such as years played, hours spent per week, and team/individual achievements.
