# Supplementary material for: Retrospective survey of youth sports participation: Development and assessment of reliability using school records
Source: PLoS One. 2021 Sep 17;16(9):e0257487. doi: 10.1371/journal.pone.0257487 (PMC8448309; doi:10.1371/journal.pone.0257487)
Supplement: Attachment — Submitted filename: Response to Reviewers.docx [file pone.0257487.s006.docx]

**Retrospective survey of youth sports participation: development and validation using school records**

**Manuscript ID:**

**POINT-BY-POINT RESPONSE**

We thank the two anonymous reviewers for their helpful comments on the revision of our submitted manuscript. We have addressed each of these comments in our revised manuscript and have provided a point-by-point response below. Note that, in some cases, reviewer comments have been combined, shortened, or separated into comments with a single theme. Reviewer comments are **bold-faced** whereas our responses are in normal font.

**Reviewer 2 Comment 1: BIG PICTURE

The main concern is still around the use of Cronbachs alpha. If you are only looking at number of sports from two different sources, then technically you only have two items. There are concerns around using cronbach’s alpha with only two items See, Eisinga, R., Te Grotenhuis, M., & Pelzer, B. (2013). The reliability of a two-item scale: Pearson, Cronbach, or Spearman-Brown?. International journal of public health, 58(4), 637-642. The authors might consider only using percent agreement in this study.**

Response: We are grateful for the helpful references. We agree with the referee that Cronbach’s alpha may be inappropriate in this setting and we have removed this analysis from our revised manuscript. We now report only percent agreement between the yearbook record and survey self-report.

**Reviewer 2 Comment 2: There is a concern around the use of the word validity and validation in the study. Finding reliability does not necessarily mean that a measure is valid. It can be misleading to use the word validity when there was no measure of validity in the study. This may be incorrect, so I leave it up to the editors discretion.**

Response: Thank you for bringing up the distinction between reliability and validity. We have replaced the word “validity” with “reliability” throughout the manuscript and retitled the manuscript “The sample was a convenience sample comprised of the authors’ alumni networks, and not a representative sample of different types of schools across geographic regions.”

**Review 2 Comment 3: Survey development / Survey

I am unsure how the sports included in the study were chosen at the authors state two different reasons. “The sports included in the survey were selected for their popularity at the high school/collegiate level and the potential risk for a hit on the head or concussion” and

“The specific 15 sports (listed in Table 1) included in our survey were selected based on sports participation surveys conducted by the National Federation of State High School Associations.”

Please clarify which was the correct criteria for inclusion.**

Response: Thanks for pointing out this discrepancy. We have clarified the correct criteria for inclusion as “The specific 15 sports (listed in Table 1) included in our survey were selected based on sports participation surveys conducted by the National Federation of State High School Associations as well as knowledge of other sports that were popular at the schools where the pilot study described in the next section was conducted.”

**Reviewer 2 Comment 4: Statistical analysis

Please edit this sentence it says context twice and reads awkwardly - “ In our context, high values of Cronbach’s alpha in our context indicate that the retrospective self-reports and yearbook records are roughly measuring the same construct, sports participation”**

Response: Thanks for mentioning this. We have removed this sentence because we have removed the use of Cronbach’s alpha.

**Reviewer 2 Comment 5: Discussion**

**The last sentence mentions of ‘all three metrics’ which three are you referring to? Or do you mean both metrics?**

Response: Thanks for pointing this out. We edited this sentence to “Of note, our results support the validity of self-reported retrospective assessment of the highest frequency and highest concussion risk sports evaluated in the present sample. The self-reported retrospective assessment of football, basketball, soccer and wrestling participation all demonstrated excellent agreement with participation recorded in school yearbooks”

**Reviewer 2 Comment 6: Limitations
I do not know what you mean by “validity sample” in the sentence “ The validity sample was a convenience sample comprised of the authors’ alumni networks”**

Response: Thanks for bringing this up. We removed the word “validity” so that the sentence is now “The validity sample was a convenience sample comprised of the authors’ alumni networks, and not a representative sample of different types of schools across geographic regions.”

**Reviewer 2 Comment 7: Future directions

Again the use of validity seems misleading ‘Limitations not withstanding, the present study supports the validity of retrospective self-reported sports participation, particularly in the highest frequency, highest concussion risk sports.”

I believe you supported the reliability of the measure?**

Response: Thanks for bring this up. We have replaced “validity” with “reliability” in the sentence so it now reads: “Limitations not withstanding, the present study supports the reliability of retrospective self-reported sports participation, particularly in the highest frequency, highest concussion risk sports.”

**---------------------------------------------------------------------------------------------------------------------**

**Reviewer 3 Comment 1: Thank you for responding to reviewers' recommendations thoroughly. I do think that in the absence of actual longitudinal studies, this retrospective method with some improvements could fill the current gap. I would remove all associations with contact sport reference, and keep it broad and just concentrate on validation of the survey as a method, confirming that long term sport participation need to be investigated for its benefits too, precisely because of the large drop in participation observed in recent years.**

Response: We have removed the paragraph reviewing the literature on contact sports and risk of traumatic brain injury. Now the introduction is more broadly focused on the long-term implications of sports participation.

**Reviewer 3 Comment 2: Just a small issue of typos - participants' not participant's. Check manuscript throughout.**

Response: Thanks for pointing this out. We have replaced participant’s with participants’ throughout the manuscript.
